# The effect of pneumococcal conjugate vaccines on otitis media from 2005 to 2013 in children aged ≤5 years: a retrospective cohort study in two Swedish regions

**DOI:** 10.1080/21645515.2020.1775455

**Published:** 2020-06-23

**Authors:** Mark Edmondson-Jones, Therese Dibbern, Marcus Hultberg, Bengt Anell, Emma Medin, Yang Feng, Carla Talarico

**Affiliations:** a Parexel International, Stockholm, Sweden; b GSK, Wavre, Belgium; c GSK, Rockville, MD, USA

**Keywords:** Otitis media, Sweden, pneumococcal conjugate vaccine, incidence, time-series, age-period-cohort

## Abstract

Seven-valent pneumococcal conjugate vaccine (PCV7) was introduced to Sweden in 2009 and replaced by pneumococcal non-typeable *Haemophilus influenzae* protein D conjugate vaccine (PHiD-CV) or 13-valent PCV (PCV13) from late 2009. A retrospective cohort study assessed the impact of PCVs on otitis media/acute otitis media (OM) in children aged ≤5 years (NCT02742753) living in Skåne (PCV7 then PHiD-CV) or Västra Götalandsregionen (PCV7 then PCV13) between 2005 and 2013 using linked regional and national databases. Time-series analyses described differences between pre-PCV and post-PCV eras. Adjusted age-period-cohort (APC) predictive models estimated vaccine effectiveness and OM incidence ratios between PCV cohorts. Time-to-first OM diagnosis was estimated in ≤2 year-olds by survival analysis using a Cox proportional hazards model. Descriptive interrupted time-series analyses showed OM incidence in ≤2 year-olds declined by 42% (Skåne) and 25% (Västra Götalandsregionen) after PHiD-CV/PCV13, respectively, versus pre-PCV, but baseline OM incidence and duration of PCV7 use differed between regions. In adjusted APC models, OM incidence decreased after PHiD-CV by 9.9% (95% confidence interval [CI]: 4.4; 15.1, *p* < .001) and PCV13 by 2.3% (95%CI: −3.2; 7.6, *p* = .401) compared with pre-PCV. Both PHiD-CV and PCV13 decreased the risk of first OM diagnosis: hazard ratio (95%CI) for PHiD-CV relative to pre-PCV 0.67 (0.65; 0.69); 0.87 (0.85; 0.89) for PCV13 relative to pre-PCV; *p* < .001 for both comparisons. Within the limitations of this study conducted in two large Swedish regions, descriptive time-series analyses showed that OM incidence rates declined following the introduction of PHiD-CV and PCV13; however, this reduction only reached statistical significance for PHiD-CV in the adjusted APC models.

## Plain language summary

### What is the context?

- Otitis media, an inflammation of the middle ear, is one of the most common diseases among children worldwide. It is also one of the most frequent reasons for health-care visits and antibiotic use in young children.
- Many cases of otitis media are caused by *Streptococcus pneumoniae*, the same bacteria responsible for pneumonia and meningitis.
- The pneumococcal conjugate vaccines *Prevenar, Synflorix*, and *Prevenar 13* have been very successful in preventing invasive disease caused by *S. pneumoniae*.
- Limited data suggest that pneumococcal conjugate vaccines also decrease the incidence of otitis media, but further evidence is needed to quantify this effect.

### What is new?

- In this study, we estimated the incidence of otitis media over time, starting before the introduction of higher-valent pneumococcal conjugate vaccines (2005–2008) to well after (2009–2013). The study was conducted in two regions in Sweden, where comprehensive information on vaccine coverage and health-care utilization is available. One region used *Prevenar* then *Synflorix*; the other used *Prevenar* then *Prevenar 13*.
- Within the limitations of our study assessing vaccine impact in two large Swedish regions, our analyses suggest that:
  - The introduction of *Synflorix* and *Prevenar 13* vaccinations decreased:
    - The incidence rate of otitis media, based on descriptive time-series analysis
    - The risk of first otitis media in young children
  - Only *Synflorix* vaccination led to a significant reduction of the disease, based on a statistical model that adjusted for time, cohort, and age effects.

### What is the impact?

- This study provides further evidence that pneumococcal conjugate vaccines reduce the incidence of otitis media and delay the first episode of otitis media in infants and young children.

## Introduction

Otitis media/acute otitis media (OM) is one of the most common diseases among children worldwide, and the incidence is highest between 1 and 4 years of age.^1^ In developed countries, OM is the most frequent reason for health-care visits and antibiotic use in young children.^2,3^ The prevalence of OM varies between countries, but epidemiologic studies conducted prior to the development of pneumococcal conjugate vaccines (PCVs) found that approximately 80% of the children had experienced at least one episode of OM by the age of 3 years, with the majority of ambulatory physician visits resulting in an antibiotic prescription.^2,4-6^ The causative bacterial species identified in up to 70% of the clinical cases of OM are *Streptococcus pneumoniae*, non-typeable *Haemophilus influenzae* (NTHi), and *Moraxella catarrhalis*.^7^ Respiratory viruses such as respiratory syncytial virus and rhinovirus also play a key role in the pathogenesis of OM, and co-infections of viruses and bacteria have a synergistic effect on increasing the risk of developing OM.^8,9^

PCVs have greatly reduced the global burden of invasive pneumococcal disease due to vaccine serotypes, pneumococcal pneumonia, and all-cause pneumonia in children.^10,11^ The vaccine impact on OM has been more difficult to establish. Systematic reviews that considered the impact of the 7-valent PCV (PCV7, *Prevenar*, Pfizer; containing pneumococcal capsular polysaccharides of serotypes 4, 6B, 9V, 14, 18C, 19F, and 23F conjugated to CRM197 protein) on OM showed only modest beneficial effects of vaccination on OM incidence, mainly in low-risk children.^12,13^ However, some studies showed declines in OM beginning prior to the introduction of PCV7, and limitations in study design did not allow differentiation of the contribution of vaccination versus other factors.^13^ A more recent review of clinical trials and observational studies using higher valency PCVs (pneumococcal NTHi protein D conjugate vaccine [PHiD-CV, *Synflorix*, GSK] or 13-valent PCV [PCV13, *Prevenar 13*, Pfizer]), noted that there is evidence that these PCVs may have a greater impact on OM incidence and on antibiotic use than PCV7, although estimates of that impact vary widely due to underlying differences between studies in epidemiology, study methodology, and local OM clinical management guidelines and practices.^14^ PHiD-CV contains capsular polysaccharides of 10 pneumococcal serotypes: 1, 4, 5, 6B, 7F, 9V, 14, and 23F conjugated to NTHi protein D, 18C to tetanus toxoid and 19F to diphtheria toxoid and also induces protection against cross-reactive serotype 19A.^15^ PCV13 contains capsular polysaccharides of 13 serotypes: 1, 3, 4, 5, 6A, 6B, 7F, 9V, 14, 18C, 19A, 19F, and 23F conjugated to CRM197 protein.^16^ Because eight of the 10 serotypes in the PHiD-CV formulation are conjugated to NTHi protein D, it has been hypothesized that PHiD-CV may also protect against NTHi, and therefore could potentially have a greater impact on the incidence of OM compared to other PCVs.^17^

PCV7 was introduced into the Swedish national immunization program in 2009 and was replaced from late 2009 by PHiD-CV or PCV13, depending on the choice of the regional council. PCVs are administered in Sweden at 3, 5, and 12 months of age, and approximately 97% of the children are fully vaccinated with three doses.^18^ Studies estimating the impact of PCVs in Sweden include a randomized controlled trial, in which a 26% decrease in OM episodes in young children at risk of developing recurrent OM was observed after PCV7 introduction.^19^ An observational study conducted in the region of Skåne compared bacterial isolates from patients with an upper respiratory tract infection prior to (2007–2008) and after (2011–2013) the introduction of PCV7 and PHiD-CV.^20^ The proportion of PHiD-CV vaccine serotypes among culture-verified upper respiratory tract *S. pneumoniae* isolates decreased from 45% to 12% during the post-vaccination observation period. Although the proportion of non-PHiD-CV vaccine serotypes increased during the same period (from 49% to 80%), there was an overall reduction of 35% in pneumococcal isolates after PCV introduction and a 32% decrease in pneumococcal isolates from cases of complicated OM (recurrent or treatment failure).^20^ A retrospective study conducted in Västerbotten region compared rates of all-cause OM pre-PCV vaccination (2005–2008) with post-PCV vaccination (2014). In Västerbotten, PCV7 was replaced by PCV13 in 2010 and by PHiD-CV in 2011.^21^ Five years after the introduction of PCV, all-cause OM decreased by 41.5% and antibiotic prescriptions for bacterial OM decreased by 46.4% among ≤4 year-olds.^21^ A nation-wide observational study using the Swedish National Board of Health and Welfare (NBHW, Socialstyrelsen) database between 2005 and 2014 showed a 39% decrease in outpatient OM diagnoses and a 42% decrease in hospital admissions for OM after PCV introduction.^22^ The authors concluded that while a potentially higher impact of PHiD-CV versus PCV13 was observed in this study, it was not possible to untangle vaccine effects from preexisting epidemiological differences between the regions using the two vaccines.

Sweden is one of the only countries in the world that introduced PCVs sequentially and with high coverage and introduced higher-valent PCVs in parallel per region, thus mimicking a natural “randomized cluster design.” In this study, we used linked national and regional-level data to perform an ecologic (time-series) and individual-level approach to assess the impact of PCVs on OM in children ≤5 years of age. We took advantage of the unique Swedish setting and conducted a retrospective observational, cohort study in two regions in Sweden: Skåne (which used PCV7 from 1 January 2009 and PHiD-CV from 1 June 2010) and Västra Götalandsregionen (VGR, PCV7 from 1 January 2009 and PCV13 from 1 January 2010). Unlike prior investigations of OM in Sweden, we used three complementary statistical approaches to provide a comprehensive and nuanced analysis of the potential impact and effectiveness of PCVs on OM. An interrupted time-series model evaluated the population-level change in the incidence of OM over the three periods, individual-level Poisson modeling of age, period, and cohort (APC) effects estimated vaccine effectiveness between pairwise periods, and a time-to-event analysis evaluated time to the first OM diagnosis.

## Methods

### Study objectives

The primary study objective was to assess the impact of PCVs by describing separately for Skåne and VGR trends over time in the incidence of OM among children during pre-PCV, PCV7, and PHiD-CV/PCV13 vaccination eras. The secondary study objectives reported here were assessment of severe OM over the same vaccination eras, possible individual-level predictors of OM rates, and impact of vaccination on time-to-first OM diagnosis. Other secondary objectives, namely, rates of antibiotic use and tympanostomy tube placement, and estimates of OM-related direct costs and health-care resources used in each region, will be reported separately.

### Feasibility assessment

All 21 regional councils in Sweden were included in an extensive feasibility assessment to determine if their health-care registries could be used to achieve the study objectives (see details in the Supplement). Ten regions were ruled out due to population size and vaccine usage, and three more were ruled out because they introduced PCVs as late as 2011 and therefore did not allow sufficiently long follow-up. Only three of the remaining regional councils had administrative databases with data on vaccinations, diagnoses, and procedures which were collected on an annual basis from electronic medical records at public and private hospitals, pediatric health-care centers, and primary care centers. Skåne and VGR were identified as suitable to include in the study, based on the information collected in their regional council administrative databases, available data about vaccine use, similar years of PCV13 or PHiD-CV introduction, similar population sizes, and similarities in demographic features (e.g., number of children, population density, number of children in day care), household characteristics (e.g., average number of persons per household, average number of children per household), and sociodemographic factors (e.g., income and unemployment).

Together, Skåne and VGR residents represent 30% of the entire Swedish population. Both regions provide electronic databases linkable at a patient-level to national registries. The populations in these regions were similar to Swedish national averages in terms of number of individuals living together in households, average number of children per person, average individual annual income, unemployment rates, exposure to tobacco smoke, proportion of children aged ≤3 years in day care, and vaccine coverage.

### Study design

All children born in Skåne and VGR from 1 January 1999 through 31 December 2013 were identified from the Swedish Medical Birth Registry. Children with outcomes of interest were identified and assigned to PCV cohorts according to their date and region of birth, with respect to the implementation of PCV7, PHiD-CV, and PCV13 (Table 1). The pre-PCV cohort was evenly divided between ≤2 and 3–5 year-olds, whereas the PHiD-CV and PCV13 cohorts primarily represented ≤2 year-olds. All children were assumed to be fully vaccinated. A 3-month “washout period” was defined after the implementation of each new vaccine. Children born within the “washout periods” were not included in cohort comparisons, but were included in the calculation of monthly and annual OM incidence, and corresponding time-series analyses.

**Table 1.** Assignment of children to vaccination cohorts according to date and region of birth

Cohort	Region of birth	Date of birth range*
pre-PCV	Either	1 January 1999–31 December 2008
PCV7	VGR	1 April 2009–31 December 2009
	Skåne	1 April 2009–31 May 2010
PCV13	VGR	1 April 2010–31 December 2013
PHiD-CV	Skåne	1 September 2010–31 December 2013

PCV = pneumococcal conjugate vaccine, PCV7/PCV13 = 7-valent/13-valent pneumococcal conjugate vaccine, PHiD-CV = pneumococcal non-typeable *Haemophilus influenzae* protein D conjugate vaccine, VGR = Västra Götalandsregionen

*the assignment of children to each cohort includes a 3-month washout period following vaccine implementation

Children with urinary tract infection (UTI) diagnosed either in primary care or as an inpatient or outpatient were used as an indicator control disease for the time-series analyses.

The study was conducted in accordance with applicable regulatory requirements, including applicable subject privacy requirements and the guiding principles of the Good Epidemiological Practice and the Declaration of Helsinki. Before the collection of the study data, approval of the protocol was obtained from the Central Ethical Review Board in Sweden. The study was registered on ClinicalTrials.gov (NCT02742753).

### Data sources

All pregnancies and births in Sweden, including pregnancy duration, weight, length, and health status of the infant at delivery, type of delivery, family constitution, and the mother’s civil status and tobacco habits are recorded in the Medical Birth Registry. The Registry was used to capture birth-related variables considered in the individual-level predictive analysis for the incidence of OM.

The National Patient Registry (Patientregistret) is held by the NBHW and captures patient characteristics including demography, information on inpatient and outpatient episodes (primary and secondary diagnoses), and procedures. Data collection is mandatory and the register is updated annually from all hospitals and outpatient clinics by the NBHW. The under-reporting rate was estimated to be <1% for inpatient visits and close to 4% for specialized outpatient care, and as such it has close to complete coverage of health-care-related information on the Swedish population.^23,24^

The Region West Health-care Database (Vårddatabasen Vega) and the Patient Administrative System in the region of Skåne (Patientadministrativt system i Skåne) are the regional health-care databases for VGR and Skåne, respectively. These databases contain patient-level data on diagnoses and procedures from primary health-care providers (private and public) in their regions.

Cases of OM were retrieved from the National Patient Registry and the regional primary care data sources. Data from the local registries were merged with data from the national registries at the patient level with the support of the NBHW, and de-identified data were transferred with unique identifiers. The analysis was conducted by Parexel International, which is registered with the Swedish Data Protection Authority.

### Variables and clinical definitions

Primary and secondary diagnoses of OM were identified using the 10^th^ revision of the International Classification of Diseases (ICD-10) code H66 (suppurative and unspecified OM) and H65 (non-suppurative OM). As defined in other studies of OM,^25^ an OM diagnosis was considered as a new diagnosis if a child presented with OM 14 days or more after a previous OM episode. Severe OM was defined as ≥3 diagnoses of OM in a six-month period or ≥4 OM diagnoses in a 12-month period, or hospitalization due to OM. Analytic rules were introduced to avoid double counting of patients with any registered diagnosis of OM in both the National Patient Registry and the regional data sources occurring within two weeks of another (i.e., to avoid double counting of an OM case in situations where the same case is captured by the primary health-care system and hospital system). Details of comorbidities associated with increased risk of invasive pneumococcal disease were provided by NBHW (Table S1).

The UTI control indicator diagnosis for the time-series analyses was defined as ICD-10 code N39.

### Statistical methods

Statistical analyses were performed using R version 3.4.2. The analyses were conducted starting with a descriptive summary followed by time-series analysis. Analyses were performed separately for each region and no direct statistical comparisons between the two regions were performed. The analytic period was between 1 January 2005 and 31 December 2013 and began six years after the birth data capture began, in order to allow children to be fully represented across the age range. The study period ended on 31 December 2013 because Skåne switched from PHiD-CV to PCV13 in May 2014. Incidence of OM was not reported separately per diagnostic code due to potential clinical ambiguity between diagnoses.

Crude monthly OM incidence rates were obtained by dividing the number of OM cases each month by the total at risk population that month. The total at risk population was estimated based on the total number of days from birth, as recorded in the Medical Birth Registry, to the earlier of death, as recorded in the Cause of Death Registry and censored at the date of death, or attainment of age six, that fall within each calendar month per region and age group. In/out migration data were not available for the regional councils.

Sensitivity analyses were performed in order to assess the effect of different PCV “washout periods” in the regions, and the potential effects of assuming 100% PCV coverage (Supplement).

#### Time-series analysis

To investigate the temporal dynamics (seasonality and autocorrelation) in monthly/annual incidence for each outcome in Skåne and VGR separately, and to compare the monthly incidence rates between the pre-PCV, PCV7, and PHiD-CV/PCV13 cohorts in each region, an autoregressive integrated moving average regression analysis was performed using three primary stages according to the Box–Jenkins methodology.^26^

Interrupted time-series analyses were used to detect whether the introduction of a vaccine had a significantly greater effect on the incidence of OM, accounting for any underlying secular trends. The time-series were divided into three time periods; the pre-PCV vaccination era, PCV7 era, and PHiD-CV/PCV13 era. Segmented regression analysis was used to measure the changes of incidence of OM in level and slope between the different vaccination eras, allowing for the transition periods. These were modeled by allowing each month’s incidence to be independently estimated, thereby not introducing a break in the time-series which would make the estimation of autocorrelation and seasonality problematic. These models were fitted using the following formula:

$$Incidence\;=\;\left(\alpha_{1}+\beta_{1}t\right)+PCV\left(\alpha_{2}+\beta_{2}\left(t-t_{2}\right)\right)+PCV13\left(\alpha_{3}+\beta_{3}\left(t-t_{3}\right)\right)$$

where *t* is the month number, *PCV* is an indicator set to 1 if in the PCV7 or PHiD-CV/PCV13 eras, or 0 otherwise, and where *PCV13* is an indicator set to 1 if in the PHiD-CV/PCV13 eras or 0 otherwise. Indicators *PCV* and *PCV13* are both delayed for the 3-month transition period to reflect the time taken for the vaccines to be implemented and take effect. *t_2_* and *t_3_* indicate the month numbers when PCV7 and PHiD-CV/PCV13 were introduced, following the 3-month transition period. The initial level of incidence (at time *t* = 0) is represented by *α_1_*, and the change from pre-PCV to PCV7 and from PCV7 to PHiD-CV/PCV13 is represented by *α_2_* and *α_3_* respectively. The initial slope (rate of change of incidence rate) during the pre-PCV period is represented by *β_1_*, and changes in slope following the implementation of PCV7 and PHiD-CV/PCV13 compared to the pre-PCV period were estimated by *β_2_* and *β_3_*, respectively. The change in slope from the PCV7 to the PHiD-CV/PCV13 period was estimated by *β_3_ – β_2_*.

To allow for the fact that within each calendar year children may have received different vaccinations, or none, according to their year of birth (and therefore age), separate analyses were performed by age category (≤2 and 3–5 years).

#### Individual-level predictive models of OM rates and time-to-first diagnosis

Children in each PCV cohort were followed until their sixth birthday. Potential associations between possible predictors and OM rates were analyzed independently by an APC model using multivariable over-distributed Poisson regression models, attempting to account for potential PCV herd effects on the incidence of OM. The model is expressed algebraically as:

$$log\left(N\right)=X\beta +log\left(P\right)$$

where *N* and *P* are the number of cases and population at risk per stratum, with strata defined by each unique combination of covariates/factors, as described by *X. β* defines the coefficients corresponding to these covariates/factors. Within regional council comparisons between the vaccine cohorts and the pre-PCV cohorts were performed using adjusted incidence rate ratios (IRRs) with the corresponding 95% confidence intervals (CIs). Vaccine effectiveness was defined as 1 minus the IRR.

The association between time-to-first OM diagnosis in children aged ≤2 years and explanatory variables was estimated by survival analysis using a confounder-adjusted right-censored Cox proportional hazard model. This model was adjusted for sex, health condition at birth, a previous diagnosis of OM, maternal age, and maternal smoking at pre-conception and at 30 weeks of gestation. The survival analysis was described by Kaplan–Meier curves. Adjusted hazard ratios and 95% CIs are presented together with test estimates. The target group for these analyses was children ≤2 years of age because incidence of OM is highest in this age group, and this age group was fully represented in all three cohorts.

## Results

### Baseline characteristics

We identified 191,596 children born in Skåne between 1999 and 2013, of whom 77,962 (41%) had ≥1 diagnosis of OM, including 60,141/123,794 (49%), 7,869/17,811 (44%), and 9,952/49,991 (20%) of children in the pre-PCV, PCV7, and PHiD-CV cohorts, respectively. Among 250,327 children born in VGR during the study period, 102,490 (41%) had ≥1 diagnosis of OM, including 77,966/165,683 (47%), 7,272/14,324 (51%), and 17,252/70,320 (25%) of children in the pre-PCV, PCV7, and PCV13 cohorts, respectively.

Sex distribution, weight and length at birth, maternal characteristics, and co-morbidities were similar between the PCV cohorts, and between children with and without a diagnosis of OM (Tables S2-S4).

### Incidence of OM

Table 2 shows the annual OM incidence rates in the total study population. The annual incidence of OM per 100,000 person-years decreased over the study period, from 30,859 in 2005 to 20,207 in 2013 in Skåne, and from 24,473 in 2005 to 21,411 in 2013 in VGR. In each study year, the annual incidence of OM was greatest among children aged ≤2 years.

**Table 2.** Yearly incidence (per 100,000 person-years) of otitis media/acute otitis media among all children according to age*

Age	2005	2006	2007	2008	2009	2010	2011	2012	2013
Skåne Incidence rate (95% CI)									
≤2 years	37,124 (36,507; 37,752)	39,868 (39,233; 40,513)	42,452 (41,808; 43,106)	42,135 (41,508; 42,772)	37,810 (37,230; 38,400)	37,041 (36,477; 37,614)	30,544 (30,038; 31,059)	37,124 (36,507; 37,752)	39,868 (39,233; 40,513)
3–5 years	24,009 (23,491; 24,538)	24,342 (23,828; 24,866)	25,369 (24,853; 25,897)	24,142 (23,645; 24,650)	21,717 (21,250; 22,195)	21,545 (21,088; 22,012)	20,109 (19,677; 20,551)	19,491 (19,075; 19,916)	16,454 (16,079; 16,837)
≤5 years	30,859 (30,451; 31,272)	32,396 (31,983; 32,814)	34,256 (33,838; 34,679)	33,573 (33,166; 33,984)	30,745 (30,355; 31,140)	29,317 (28,937; 29,701)	24,837 (24,490; 25,190)	23,918 (23,582; 24,259)	20,207 (19,901; 20,518)
VGR Incidence rate (95% CI)									
≤2 years	29,283 (28,811; 29,763)	30,836 (30,357; 31,324)	34,945 (34,441; 35,458)	34,316 (33,822; 34,817)	32,014 (31,542; 32,492)	38,730 (38,217; 39,249)	31,800 (31,338; 32,268)	29,432 (28,989; 29,882)	25,914 (25,496; 26,338)
3–5 years	19,280 (18,883; 19,686)	21,357 (20,944; 21,779)	22,990 (22,567; 23,422)	21,841 (21,434; 22,257)	19,539 (19,158; 19,928)	23,601 (23,187; 24,023)	21,320 (20,932; 21,716)	19,143 (18,779; 19,514)	16,317 (15,985; 16,655)
≤5 years	24,473 (24,161; 24,788)	26,275 (25,955; 26,598)	29,183 (28,851; 29,520)	28,304 (27,981; 28,631)	26,452 (26,137; 26,771)	31,171 (30,824; 31,521)	25,671 (25,359; 25,986)	24,227 (23,927; 24,532)	21,411 (21,130; 21,696)

CI = confidence interval, VGR = Västra Götalandsregionen

*each study year contains a different blend of unvaccinated children, versus children vaccinated with either the 7-valent pneumococcal conjugate vaccine, the 13-valent pneumococcal conjugate vaccine, or the pneumococcal non-typeable *Haemophilus influenzae* protein D conjugate vaccine

Table 3 shows OM incidence rates according to the vaccination cohort (as defined in Table 1). There was a decline in the overall incidence rate of OM from the pre-PCV cohort through the consecutive PCV cohorts for most cohorts and age groups (Table 3). This decline was most marked in the ≤2-year population in whom the annual incidence of OM decreased by 42% in the PHiD-CV cohort compared with the pre-PCV cohort (23,697 versus 40,843 per 100,000 person-years) in Skåne. In VGR, the incidence rate was 25% lower in the PCV13 cohort versus the pre-PCV cohort (25,710 versus 34,501 per 100,000 person-years). These descriptive results in children aged 3–5 and ≤5 years should be interpreted with caution considering children in the 3–5 year group were not vaccinated in the initial years of PCV7 and PHiD-CV/PCV13 periods.

**Table 3.** Overall incidence per 100,000 person-years of otitis media/acute otitis media in the pre-PCV, PCV7, and PHiD-CV/PCV13 cohorts*

Cohort	≤2 years	3–5 years	≤5 years
Skåne Incidence rate (95% CI)			
pre-PCV	40,843 (40,574; 41,113)	22,034 (21,871; 22,199)	29,721 (29,574; 29,868)
PCV7	31,469 (30,996; 31,949)	17,623 (17,061; 18,203)	27,583 (27,208; 27,964)
PHiD-CV	23,697 (23,367; 24,032)	17,994 (15,325; 21,128)	23,640 (23,312; 23,973)
VGR Incidence rate (95% CI)			
pre-PCV	34,501 (34,287; 34,717)	20,819 (20,682; 20,958)	26,376 (26,257; 26,497)
PCV7	33,099 (32,559; 33,648)	17,848 (17,269; 18,445)	28,275 (27,861; 28,694)
PCV13	25,710 (25,433; 25,990)	15,270 (14,283; 16,325)	25,268 (24,999; 25,539)

CI = confidence interval, PCV = pneumococcal conjugate vaccine, PCV7/PCV13 = 7-valent/13-valent pneumococcal conjugate vaccine, PHiD-CV = pneumococcal non-typeable *Haemophilus influenzae* protein D conjugate vaccine, VGR = Västra Götalandsregionen

*the PCV7 era was short in VGR and Skåne and any change detected vs the PCV7 cohort in the incidence of otitis media/acute otitis media should be considered with caution

Compared to the pre-PCV cohort, the incidence of severe OM in the ≤2-year population decreased by 50% in the PHiD-CV cohort and 32% in the PCV13 cohort (Table S5).

### Time-series analyses

**Figure 1.**
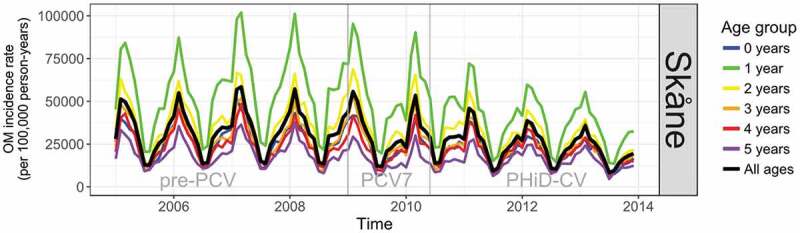
Time-series showing monthly incidence of otitis media/acute otitis media (OM) in the pre-PCV, PCV7, and PHiD-CV eras in Skåne

**Figure 2.**
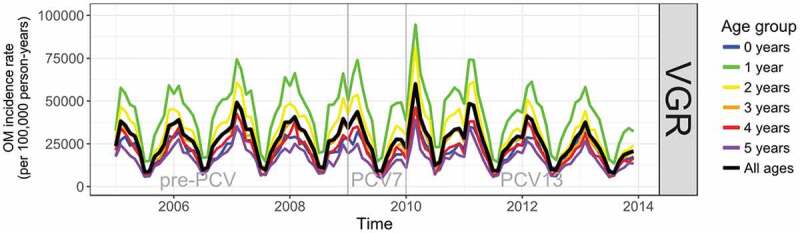
Time-series showing monthly incidence of otitis media/acute otitis media (OM) in the pre-PCV, PCV7, and PHiD-CV eras in VGR

OM incidence had a strong annual seasonality and significant levels of autocorrelation in all ages and in each vaccine era (Figures 1 and 2) peaking in February to March and with a trough in July to August. The PCV7 period in each region was too short (17 months in Skåne and 12 months in VGR) to detect any meaningful change in the incidence of OM in the time-series analyses, and results for pre-PCV versus PCV7, and PCV7 versus PHiD-CV/PCV13 periods are provided in the supplement. Before the introduction of PCVs, the incidence rate of OM in children aged ≤5 years was higher in Skåne than in VGR, particularly among children aged 1 year (Figures 1 and 2).

PCV = pneumococcal conjugate vaccine, PCV7 = 7-valent pneumococcal conjugate vaccine, PHiD-CV = pneumococcal non-typeable *Haemophilus influenzae* protein D conjugate vaccine

PCV = pneumococcal conjugate vaccine, PCV7/PCV13 = 7-valent/13-valent pneumococcal conjugate vaccine, VGR = Västra Götalandsregionen

From the interrupted time-series analysis, the annual rate of change in the incidence of OM in the PHiD-CV era relative to the pre-PCV era was statistically significantly different in Skåne for all years of age under 3 years of age (Figure S1A and Table S6). Compared to the annual rate of change in incidence in the pre-PCV-era, the incidence per 100,000 person-years in the PHiD-CV era was lower by 365 (95% CI: 38; 692) per annum (pa) in <1 year-olds, 820 (95% CI: 237; 1,403) pa in 1 year-olds, and 552 (95% CI: 200; 904) pa in 2-year-old children. The reduction over all ages (≤5 years) was 382 (95% CI: 123; 641) events pa per 100,000 person-years. In VGR the rate of change in the incidence of OM per 100,000 person-years in the PCV13 era relative to the pre-PCV era was statistically significant for all years of age; 185 (95% CI: 51; 318) pa lower in <1 year-olds, 825 (95% CI: 404; 1,246) pa in 1 year old children, 664 (95% CI: 341; 988) pa in 2 year-olds, 425 (95% CI: 193; 657) pa in 3 year-olds, 289 (95% CI: 91; 487) pa in 4 year-olds, and 197 (95% CI: 31; 363) pa in 5 year-olds. The overall reduction in the rate of change in incidence (≤5 years) was 436 (95% CI: 221; 651) events pa per 100,000 person-years (Figure S2A and Table S6).

The reduced incidence of OM was maintained under the sensitivity analysis where the “washout period” was extended to be equal in both regions (Figures S1B and S2B and Table S7). Further, the estimated incidence assuming 100% coverage was very close to that observed in the sensitivity analysis using officially reported coverage rates (Table S8).

For severe OM, strong seasonality and autocorrelation were apparent (Figures S3 and S4). The interrupted time-series showed a consistent reduction in both regions from the pre-PCV to the PHiD-CV/PCV13 eras in most age groups and overall.

UTI was used as a control indicator disease for the time-series analyses. There was no evidence of seasonality, autocorrelation, or systematic trends in the incidence of UTI observed in the interrupted time-series analysis.

### Individual-level predictive models for incidence of OM

Higher incidence rates of OM were associated with male sex, a previous diagnosis of OM, and winter season, while lower rates were associated with increasing age from the second birthday onwards (≤5 year population, Table S9). The strongest correlation with OM was a previous OM diagnosis. One previous OM diagnosis was associated with an increase in the incidence rate by 2.7–4.6 times as compared to children with no previous diagnosis, and two or more previous diagnoses of OM increased the incidence rate still further, up to 5.5–10.0 times the rate of OM as compared to children with no previous diagnosis. These findings were observed consistently in both regions and in all three vaccine cohorts (Table S9).

In Skåne, the adjusted incidence rate in children ≤5 years for OM was significantly lower in the PHiD-CV cohort than the pre-PCV cohort (IRR 0.901, 95% CI: 0.849; 0.956) (Table 4). There were no significant changes in the incidence of OM in VGR after PCV13 was introduced compared to the pre-PCV cohort. It follows that vaccine effectiveness in preventing OM relative to the pre-PCV cohort was 9.9% (95% CI: 4.4; 15.1; *p* < .001) for PHiD-CV and 2.3% (95% CI: −3.2; 7.6; *p* = .401) for PCV13.

**Table 4.** Incidence rate ratios of otitis media/acute otitis media diagnoses between vaccine cohorts adjusted for confounding variables, according to the age-period-cohort Poisson model

	Skåne			VGR		
PCV Cohorts	IRR	95% CI	p-value	IRR	95% CI	p-value
≤5 years of age						
PHiD-CV/PCV13 relative to pre-PCV	0.901	0.849; 0.956	**<0.001**	0.977	0.924; 1.032	0.401
PHiD-CV/PCV13 relative to PCV7	0.926	0.884; 0.970	**0.001**	1.017	0.969; 1.067	0.493
PCV7 relative to pre-PCV	0.973	0.932; 1.015	0.207	0.961	0.917; 1.006	0.087
≤2 years of age						
PHiD-CV/PCV13 relative to pre-PCV	0.952	0.866; 1.047	0.310	0.952	0.874; 1.037	0.263
PHiD-CV/PCV13 relative to PCV7	0.967	0.904; 1.035	0.330	0.994	0.930; 1.063	0.871
PCV7 relative to pre-PCV	0.985	0.927; 1.046	0.614	0.958	0.901; 1.018	0.164

CI = confidence interval, IRR = incidence rate ratio, PCV = pneumococcal conjugate vaccine, PCV7/PCV13 = 7-valent/13-valent pneumococcal conjugate vaccine, PHiD-CV = pneumococcal non-typeable *Haemophilus influenzae* protein D conjugate vaccine, VGR = Västra Götalandsregionen

The APC predictive Poisson models were run separately for the ≤2 year age group, as this age group was vaccinated in both the PCV7 and PHiD-CV/PCV13 cohorts. The predictive factors were found to be broadly consistent with those estimated for all children aged ≤5 years old (data not shown). However, the comparisons between the higher-valent vaccine cohorts and the pre-PCV cohort were not statistically significant when this smaller age range was analyzed (Table 4).

### Time-to-first OM diagnosis in children ≤2 years of age

The time-to-event analysis showed that after the introduction of PCVs, the time-to-first OM diagnosis increased in children *≤* 2 years of age in both regions (Figures S5 and S6). PHiD-CV and PCV13 vaccinations were associated with a decrease in the risk of first OM diagnosis relative to pre-PCV; adjusted hazard ratio 0.673 (95% CI: 0.654; 0.692) and 0.867 (95% CI: 0.849; 0.886) for PHiD-CV and PCV13 relative to pre-PCV, respectively; *p* < .001 for both comparisons (Table S10).

## Discussion

We used real-world data from mandatory national Swedish health-care registries and both population and individual-level statistical approaches to estimate the impact of PCVs on OM over time. The burden of OM was considerable in all age groups, with 41% of the children aged ≤5 years having experienced at least one OM episode during the study period. While the PCV7 era was too short in VGR and Skåne to detect any change in the incidence of OM in the time-series analyses, a clear reduction in OM incidence, including severe OM, was observed after the introduction of higher-valent PCVs. The incidence of OM among ≤2 year-olds decreased by 42% in Skåne in the PHiD-CV cohort relative to the pre-PCV cohort, and by 25% in VGR in the PCV13 cohort relative to the pre-PCV cohort. The results were maintained under a sensitivity analysis whereby the higher-valent vaccine transition period was extended across both regions to exclude a consistent period that spanned the implementation of both vaccines. Similar decreases were observed for severe OM (50% and 32% decrease in the PHiD-CV and PCV13 cohorts, respectively, compared to the pre-PCV cohort).

Compared to the pre-PCV era, vaccine effectiveness in preventing OM in ≤5 year-olds was 9.9% for PHiD-CV and 2.3% for PCV13. The adjusted APC Poisson model suggested that only the PHiD-CV cohort was associated with a statistically significant reduction in OM incidence. In terms of age, the pre-PCV cohort was evenly divided between the ≤2 and 3–5 age groups. Conversely, few children in the PHiD/PCV13 cohorts were followed up beyond their third birthday and these cohorts were thus mostly comprised of ≤2 year-olds. This means that in the PHiD-CV and PCV13 cohorts, the overall incidence rates are weighted toward this younger age group. However, the presence of older children within the APC analysis facilitated more accurate estimation of the time effect, and thereby more accurate isolation of the cohort effect by virtue of the APC model structure. Partly as a consequence, no significant changes were observed in the analysis of ≤2 year old children, as the older children were not present to facilitate a more accurate estimate of the time trend. However, the estimates for ≤2 year-olds were in the same direction, but their variability was correspondingly higher. The advantage of the APC model is that it provides a means of concurrently modeling the separate effects of time and cohort (potential herd effects), while controlling for confounding variables such as age; however, it assumes that the underlying model accurately describes the population–disease interaction. Allowing for over-distribution in the Poisson regression allows potential adjustment for correlation among similar ages, periods, and cohorts or the potential for unmeasured confounding.^27^

Within regional councils and across time, we observed a statistically significant reduction in the incidence rate of OM in Skåne but not in VGR, and a greater relative reduction in the risk of first OM diagnosis in Skåne in the PHiD-CV cohort relative to the pre-PCV cohort than that observed in VGR compared to the pre-PCV cohort. The potential of unmeasured confounding variables does not allow us to make direct statistical comparisons between the two regions. However, other studies have implied a greater impact of PHiD-CV on OM than PCV13. The nationwide Swedish study reported by Marie Gisselsson-Solen 2017^22^ observed greater decreases in ambulatory visits for OM and tympanostomy tube placements in regions that used PHiD-CV compared with PCV13, although large geographical differences between regions were observed prior to vaccine introduction. A differential impact on OM is perhaps not unexpected given that vaccination with PHiD-CV induces high levels of NTHi-specific (anti-protein D) antibodies, and consistent trends toward vaccine efficacy against NTHi were observed in randomized controlled trials of NTHi protein D conjugated PCVs, with estimates for PHiD-CV ranging between 15% and 22%.^17^

Strengths of the current study include the use of data from large, linked regional and national databases with high levels of coverage, allowing population-level assessment of temporal trends in OM incidence over the period before and after the introduction of different PCVs. Such studies contribute to the overall picture of PCV impact on OM and inform potential differences between the impact of these vaccines. The three different statistical approaches each allow analysis of different outcomes of interest and provide insight regarding the different ways that PCVs may be impacting pneumococcal epidemiology at the population and individual levels. We used descriptive time-series analysis to test for differences in population-level OM incidence during pre- versus post-vaccination periods and used modeling techniques to adjust the OM incidence estimates for the observed seasonality, autocorrelation, and potential confounding variables.

Potential limitations that could affect results interpretation include the assumption that PCV coverage was 100%. In view of high national vaccine coverage in Sweden, it was predicted that this assumption introduced little bias and was confirmed by the sensitivity analyses using published coverage rates. Information for some potential individual-level confounders such as pre-school/day care attendance, exposure to environmental tobacco smoke, and number of siblings living in the same household were not available. While regional-level characteristics in terms of average household size, average number of children per household, income, exposure to tobacco smoke within households, unemployment, and day care use in Skåne and VGR were similar to national averages, we cannot exclude that differences between families with and without children with an OM diagnosis may have influenced the APC analysis. In/out migration was not able to be quantified and was assumed to be negligible in the analyses. However, national statistics suggest that the level of migration is similar in Skåne and VGR so any potential bias is expected to be consistent between the regions. We cannot rule out the influence of other factors that could potentially impact OM incidence over time, such as OM misdiagnosis, changes in health-care-seeking behavior, or changes in treatment practices post-vaccine introduction, although any such effects might be expected to affect both regions similarly. Finally, approximately four years of post-PHiD-CV/PCV13 data were available for analysis, as Skåne switched from PHiD-CV to PCV13 in May 2014.^28^ It is possible that a longer period of post-PHiD-CV/PCV13 follow-up could have led to different conclusions about the impact of PCVs on all-cause OM epidemiology in Sweden. It should be noted, however, that countries who implemented higher-valent PCVs with fast uptake and high, consistent coverage (as was done in Sweden) have reported rapid decreases in OM incidence within 2–3 years of vaccine introduction, suggesting that longer periods are not needed to observe vaccine impact.^29,30^

In conclusion, within the limitations of our study assessing vaccine impact in two large Swedish regions, descriptive time-series analysis showed a decline in the incidence rate of OM following the introduction of higher valency PCVs, and cohorts defined by the sequential introduction of PCVs similarly experienced declines in OM incidence rates. Adjusted APC models suggest that only PHiD-CV was associated with a significant reduction in the incidence rate of OM. PHiD-CV and PCV13 both appeared to decrease the risk of first OM in young children.

## Supplementary Material

Supplemental Material

## Figures and Tables

**Table:** 

APC	age-period-cohort
CI	confidence interval
ICD-10	10^th^ revision of the International Classification of Diseases
IRR	incidence rate ratio
NBHW	Swedish National Board of Health and Welfare
NTHi	non-typeable *Haemophilus influenzae*
OM	otitis media/acute otitis media
PCV	pneumococcal conjugate vaccine
PCV7/PCV13	7-valent/13-valent pneumococcal conjugate vaccine
PHiD-CV	pneumococcal non-typeable *Haemophilus influenzae* protein D conjugate vaccine
UTI	urinary tract infection
VGR	Västra Götalandsregionen

## Trademarks

*Synflorix* is a trademark licensed to the GSK groups of companies. *Prevenar* and *Prevenar 13* are trademarks of Pfizer.
