# Supplementary material for: The effect of pneumococcal conjugate vaccines on otitis media from 2005 to 2013 in children aged ≤5 years: a retrospective cohort study in two Swedish regions
Source: Hum Vaccin Immunother. 2020 Jun 23;17(2):517–26. doi: 10.1080/21645515.2020.1775455 (PMC7899701; doi:10.1080/21645515.2020.1775455)
Supplement: Supplemental Material [file KHVI_A_1775455_SM8859.docx]

**The effect of pneumococcal conjugate vaccines on otitis media from 2005–2013 in children aged ≤5 years: a retrospective cohort study in two Swedish regions**

Mark Edmondson-Jones, Therese Dibbern, Marcus Hultberg, Bengt Anell, Emma Medin, Yang Feng, Carla Talarico

**Supplementary material**

Feasibility assessment

All 21 regional councils in Sweden were included in an extensive feasibility assessment to determine if regional (i.e., regional council) healthcare registries could be used to achieve the study objectives. Four data collection methods were used:

- Literature review
- Desk research of grey literature and data source documentation
- Interviews with experts and representatives from organizations of interest
- Interim project meetings

The first main goal of the feasibility assessment was to identify regional councils using electronic medical record systems (versus paper-based medical record systems), with which person-level data could be retrieved and linked with person-level data from national databases. The other main goal was to identify regional councils that had used pneumococcal conjugate vaccines (PCVs) for similar time periods and that had demographic and health behavior characteristics that were similar to each other and to national-level demographics. A similar demographic and health behavior profile was necessary because we had no access to potential confounders at an individual level (e.g., number of children aged <5 years within households, number of children ≤3 years attending day care, child’s exposure to tobacco smoke).

Because of herd effects caused by high and consistent use of PCVs, it was critical to select regions that used 7-valent PCV and then either pneumococcal non-typeable *Haemophilus influenzae* protein D conjugate vaccine (PHiD-CV) or 13-valent PCV (PCV13) during similar time periods. This was necessary to ensure that the epidemiology of circulating pneumococcal serotypes was similar across the regions at the beginning of the study period.

Skåne and VGR account for 30% of the total Swedish population and were the most comparable in terms of PCV use (year of introduction and coverage). Both regions had large sample sizes compared to some other regions, and had accessible and high quality electronic medical records systems in place that spanned the study period. They also maintained administrative databases (Patientadministrativt system i Skåne and Vårddatabasen Vega, respectively) holding information about vaccinations, diagnoses, and procedures from electronic medical records at public and private hospitals within the regions. Both databases could be linked at the individual level with national registries.

In 2015 there were 202 primary care centers in VGR, of which 113 (56%) were owned by the regional council and 89 (44%) by private-care providers. All of the primary care centers had a pediatric healthcare center. Nineteen hospitals offered specialist care.

In Skåne, there were 155 primary healthcare providers in 2015, of which 86 (55%) were owned by the regional council and 69 (45%) by private providers. There were 149 pediatric healthcare centers, of which 62 (42%) were privately owned and 87 (58%) were publicly owned. There were ten hospitals within the Skåne region.

Sensitivity analyses: methods

Effect of different washout periods between regions

To account for the different washout periods at the implementation of higher-valent vaccines (Västra Götalandsregionen: 1 January 2010 – 31 March 2010; Skåne: 1 June 2010 – 31 August 2010) a wider washout period which included both (1 January 2010 – 31 August 2010) was also used for the interrupted time-series analysis. This wider period mitigated against the potential biasing effect of high rates of incidence in early 2010.

Effect of full coverage assumption

To assess the potential impact of vaccination coverage, which was slightly less than 100%, the incidence of otitis media/acute otitis media amongst vaccinated children was assessed using the relationship:

*ι_o_ = γι_v_ + (1-γ) ι_u_*

Where γ is the coverage proportion, *ι_o_* is the observed incidence, *ι_v_* is the incidence amongst vaccinated children and *ι_u_* is the incidence amongst unvaccinated children. The coverage proportion and the observed incidence were available, and the incidence amongst vaccinated children (*ι_v_*) was estimated based on the assumed incidence amongst unvaccinated children (*ι_u_*), which was estimated from the pre-PCV cohort. Coverage proportions were retrospectively reported at a regional level by the Public Health Agency of Sweden.

Table S1 Related comorbidities attributed to high or medium risk of invasive pneumococcal disease

| **Risk group** | **Specific condition** | **ICD-10 code, ATC code or procedure code** |
| --- | --- | --- |
| High | Congenital/acquired immunodeficiency* | D80–D84 |
|  | Human immunodeficiency virus* | B20–B24 |
|  | Chronic renal failure* | N18, Z49, Z992 |
|  | Nephrotic syndrome* | N04 |
|  | Generalized malignancy*, Leukemia*, Lymphoma*, Hodgkin disease*, Multiple myeloma* | C00–C43, C45–C97 |
|  | Iatrogenic immunosuppression* | ATC codes: L01, L04 |
|  | Solid organ transplant* | Z940–Z944; ICD-10, procedure codes: KAS, FQ, JJC, JLE, GDG |
|  | Functional or anatomic asplenia | D57, D730, Q890; ICD-10, procedure code: JMA |
|  | Cerebrospinal fluid leak | G960, G970 |
|  | Cochlear implant | ICD-10, procedure code: DFE00 |
| Medium | Heart disease | I01, I05–I09, I11, I13, I20–I25, I27–I28, I31, I34–I37, I42–I45, I50–I51, J81, Q20–Q25 |
|  | Liver disease (chronic) | B18, B19, K70–K77 |
|  | Lung disease (chronic) | J41–J47, J60–J70, J82, J84, J92, J99, E662, J961, J969, J982, J983 |
|  | Diabetes | E10–E14; ATC code: A10 |

ICD-10 = 10^th^ revision of the International Classification of Diseases, ATC = Anatomical therapeutic chemical classification system.

*defined as immunocompromised

Table S2 Baseline sex distribution, length, and weight at birth

|  | **Skåne** | | | | | | | | **VGR** | | | | | | | |
| --- | --- | --- | --- | --- | --- | --- | --- | --- | --- | --- | --- | --- | --- | --- | --- | --- |
|  | **pre-PCV** | | **PCV7** | | **PHiD-CV** | | **Overall** | | **pre-PCV** | | **PCV7** | | **PCV13** | | **Overall** | |
|  | **OM** | **All** | **OM** | **All** | **OM** | **All** | **OM** | **All** | **OM** | **All** | **OM** | **All** | **OM** | **All** | **OM** | **All** |
| **N** | **60,141** | **123,794** | **7,869** | **17,811** | **9,952** | **49,991** | **77,962** | **191,596** | **77,966** | **165,683** | **7,272** | **14,324** | **17,252** | **70,320** | **102,490** | **250,327** |
| **Sex, n (%)** | | | | | | | | | | | | | | | | |
| Female | 28,234 (46.9) | 60,290 (48.7) | 3,590 (45.6) | 8,635 (48.5) | 4,394 (44.2) | 24,419 (48.8) | 36,218 (46.5) | 93,344 (48.7) | 36,473 (46.8) | 80,294 (48.5) | 3,255 (44.8) | 6,844 (47.8) | 7,662 (44.4) | 34,195 (48.6) | 47,390 (46.2) | 121,333 (48.5) |
| Missing | 0 (0.0) | 0 (0.0) | 0 (0.0) | 0 (0.0) | 0 (0.0) | 0 (0.0) | 0 (0.0) | 0 (0.0) | 0 (0.0) | 0 (0.0) | 0 (0.0) | 0 (0.0) | 0 (0.0) | 1 (0.0) | 0 (0.0) | 1 (0.0) |
| **Weight, g** | | | | | | | | | | | | | | | | |
| Mean (SD) | 3,501.6 (595.8) | 3,509.7 (590.8) | 3,505.2 (604.9) | 3,499.7 (597.2) | 3,526.2 (600.9) | 3,509.1 (580.5) | 3,505.2 (597.4) | 3,508.6 (588.7) | 3,519.4 (593.8) | 3,521.7 (588.6) | 3,518.0 (590.0) | 3,515.3 (583.4) | 3,529.2 (588.8) | 3,523.0 (586.3) | 3,520.9 (592.7) | 3,521.7 (587.6) |
| Missing n (%) | 246 (0.4) | 649  (0.5) | 8  (0.1) | 10  (0.1) | 10  (0.1) | 98  (0.2) | 264  (0.3) | 757  (0.4) | 239  (0.3) | 685  (0.4) | 4  (0.1) | 7  (0.0) | 12  (0.1) | 51  (0.1) | 255  (0.2) | 743 (0.3) |
| **Length, cm** | | | | | | | | | | | | | | | | |
| Mean (SD) | 50.3 (2.4) | 50.3  (2.4) | 50.6  (2.6) | 50.5  (2.6) | 50.7  (2.5) | 50.6  (2.5) | 50.4  (2.5) | 50.4  (2.5) | 50.3  (2.5) | 50.4  (2.5) | 50.3  (2.7) | 50.3  (2.7) | 50.3  (2.6) | 50.2  (2.6) | 50.3  (2.6) | 50.3 (2.6) |
| Missing n (%) | 2,082 (3.5) | 4,206  (3.4) | 267  (3.4) | 521  (2.9) | 266  (2.7) | 1,359  (2.7) | 2,615  (3.4) | 6,086  (3.2) | 1,189  (1.5) | 2,888  (1.7) | 45  (0.6) | 93  (0.6) | 104  (0.6) | 425  (0.6) | 1,338  (1.3) | 3,406 (1.4) |

PCV = pneumococcal conjugate vaccine, PCV7/PCV13 = 7-valent/13-valent pneumococcal conjugate vaccine, PHiD-CV = pneumococcal non-typeable *Haemophilus influenzae* protein D conjugate vaccine, N = number of children in the specified cohort, n (%) = number (percentage) of children, SD = standard deviation, OM = children with one or more otitis media/acute otitis media diagnosis within the study period, All = full population regardless of otitis media/acute otitis media diagnosis, VGR = Västra Götalandsregionen

Table S3 Baseline characteristics of the child’s mother

|  | **Skåne** | | | | | | | | **VGR** | | | | | | | |  |
| --- | --- | --- | --- | --- | --- | --- | --- | --- | --- | --- | --- | --- | --- | --- | --- | --- | --- |
|  | **pre-PCV** | | **PCV7** | | **PHiD-CV** | | **Overall** | | **pre-PCV** | | **PCV7** | | **PCV13** | | **Overall** | |  |
|  | **OM** | **All** | **OM** | **All** | **OM** | **All** | **OM** | **All** | **OM** | **All** | **OM** | **All** | **OM** | **All** | **OM** | **All** |  |
| **N** | **60,141** | **123,794** | **7,869** | **17,811** | **9,952** | **49,991** | **77,962** | **191,596** | **77,966** | **165,683** | **7,272** | **14,324** | **17,252** | **70,320** | **102,490** | **250,327** |  |
| **Maternal age, years** | | | | | | | | | | | | | | | | |  |
| Mean (SD) | 29.8 (5.1) | 30.0 (5.1) | 30.1 (5.2) | 30.2 (5.3) | 30.0 (5.2) | 30.2 (5.3) | 29.9 (5.1) | 30.1 (5.2) | 29.9 (5.0) | 30.0 (5.0) | 30.3 (5.2) | 30.3 (5.2) | 30.1 (5.2) | 30.3 (5.2) | 30.0 (5.1) | 30.1 (5.1) |  |
| Missing n (%) | 0 (0) | 0 (0) | 0 (0) | 0 (0) | 0 (0) | 0 (0) | 0 (0) | 0 (0) | 0 (0) | 0 (0) | 0 (0) | 0 (0) | 1 (0.0) | 3 (0.0) | 1 (0.0) | 3 (0.0) |  |
| **Gestational length, weeks** | | | | | | | | | | | | | | | | |  |
| Mean (SD) | 39.2 (2.0) | 39.3 (2.0) | 39.2 (2.1) | 39.2 (2.0) | 39.2 (2.0) | 39.3 (1.9) | 39.2 (2.0) | 39.3 (2.0) | 39.3 (2.0) | 39.4 (2.0) | 39.4 (2.0) | 39.4 (2.0) | 39.3 (2.0) | 39.3 (2.0) | 39.3 (2.0) | 39.4 (2.0) |  |
| Missing n (%) | 19  (0.0) | 48  (0.0) | 1  (0.0) | 3  (0.0) | 1  (0.0) | 7  (0.0) | 21  (0.0) | 58  (0.0) | 13  (0.0) | 42  (0.0) | 1  (0.0) | 1  (0.0) | 1  (0.0) | 8  (0.0) | 15  (0.0) | 51  (0.0) |  |
| **Healthy child at birth, n (%)** | | | | | | | | | | | | | | | | | |
| Healthy child | 44,421 (73.9) | 92,657 (74.8) | 6,072 (77.2) | 14,036 (78.8) | 7,463 (75.0) | 38,297 (76.6) | 57,956 (74.3) | 144,990 (75.7) | 62,760 (80.5) | 135,515 (81.8) | 5,851 (80.5) | 11,666 (81.4) | 13,859 (80.3) | 47,988 (68.2) | 82,470 (80.5) | 195,169 (78.0) |  |
| Other diagnosis | 15,293 (25.4) | 30,301 (24.5) | 1,545 (19.6) | 3,263 (18.3) | 2,000 (20.1) | 10,187 (20.4) | 18,838 (24.2) | 43,751 (22.8) | 14,554 (18.7) | 28,935 (17.5) | 1,280 (17.6) | 2,413 (16.8) | 3,129 (18.1) | 20,000 (28.4) | 18,963 (18.5) | 51,348 (20.5) |  |
| Missing | 427  (0.7) | 836  (0.7) | 252  (3.2) | 512  (2.9) | 489  (4.9) | 1,507 (3.0) | 1,168 (1.5) | 2,855 (1.5) | 652  (0.8) | 1,233 (0.7) | 141  (1.9) | 245  (1.7) | 264  (1.5) | 2,332 (3.3) | 1,057 (1.0) | 3,810 (1.5) |  |
| **Family situation, n (%)** | | | | | | | | | | | | | | | | | |
| Living with children’s father | 55,107 (91.6) | 113,116 (91.4) | 7,197 (91.5) | 16,327 (91.7) | 8,920 (89.6) | 45,033 (90.1) | 71,224 (91.4) | 174,476 (91.1) | 70,214 (90.1) | 148,613 (89.7) | 6,423 (88.3) | 12,708 (88.7) | 15,141 (87.8) | 61,806 (87.9) | 91,778 (89.5) | 223,127 (89.1) |  |
| Single mother | 1,552 (2.6) | 3,380  (2.7) | 181  (2.3) | 372  (2.1) | 279  (2.8) | 1,230  (2.5) | 2,012  (2.6) | 4,982  (2.6) | 1,412  (1.8) | 3,343  (2.0) | 142  (2.0) | 256  (1.8) | 441  (2.6) | 1,480  (2.1) | 1,995 (1.9) | 5,079 (2.0) |  |
| Other family situation | 2,116 (3.5) | 4,142  (3.3) | 374  (4.8) | 850  (4.8) | 412  (4.1) | 2,175  (4.4) | 2,902  (3.7) | 7,167  (3.7) | 2,415  (3.1) | 5,143  (3.1) | 282  (3.9) | 527  (3.7) | 794  (4.6) | 3,027  (4.3) | 3,491 (3.4) | 8,697 (3.5) |  |
| Missing | 1,366 (2.3) | 3,156  (2.5) | 117  (1.5) | 262  (1.5) | 341  (3.4) | 1,553  (3.1) | 1,824  (2.3) | 4,971  (2.6) | 3,925  (5.0) | 8,584  (5.2) | 425  (5.8) | 833  (5.8) | 876  (5.1) | 4,007  (5.7) | 5,226 (5.1) | 13,424 (5.4) |  |
| **Single birth, n (%)** | | | | | | | | | | | | | | | | | |
| Singleton | 58,302 (96.9) | 119,986 (96.9) | 7,634 (97.0) | 17,277 (97.0) | 9,681 (97.3) | 48,597 (97.2) | 75,617 (97.0) | 185,860 (97.0) | 75,807 (97.2) | 160,812 (97.1) | 7,056 (97.0) | 13,913 (97.1) | 16,815 (97.5) | 68,441 (97.3) | 99,678 (97.3) | 243,166 (97.1) |  |
| **Maternal smoking 3 months before pregnancy, n (%)** | | | | | | | | | | | | | | | | | |
| Non-smoking | 44,623 (74.2) | 90,352 (73.0) | 6,259  (79.5) | 14,402  (80.9) | 7,811  (78.5) | 40,349  (80.7) | 58,693  (75.3) | 145,103  (75.7) | 56,915  (73.0) | 118,847  (71.7) | 5,596  (77.0) | 11,292  (78.8) | 13,490  (78.2) | 56,219  (79.9) | 76,001 (74.2) | 186,358 (74.4) |  |
| 1–9 cig/day | 5,853 (9.7) | 11,151 (9.0) | 676  (8.6) | 1,460  (8.2) | 834  (8.4) | 3,787  (7.6) | 7,363  (9.4) | 16,398  (8.6) | 7,085  (9.1) | 14,348  (8.7) | 606  (8.3) | 1,091  (7.6) | 1,397  (8.1) | 5,207  (7.4) | 9,088 (8.9) | 20,646 (8.2) |  |
| 10+ cig/day | 7,360 (12.2) | 13,529 (10.9) | 756  (9.6) | 1,556  (8.7) | 981  (9.9) | 4,260  (8.5) | 9,097  (11.7) | 19,345  (10.1) | 8,484  (10.9) | 16,221  (9.8) | 674  (9.3) | 1,164  (8.1) | 1,514  (8.8) | 4,966  (7.1) | 10,672 (10.4) | 22,351 (8.9) |  |
| Missing | 2,305 (3.8) | 8,762 (7.1) | 178  (2.3) | 393  (2.2) | 326  (3.3) | 1,595  (3.2) | 2,809  (3.6) | 10,750  (5.6) | 5,482  (7.0) | 16,267  (9.8) | 396  (5.4) | 777  (5.4) | 851  (4.9) | 3,928  (5.6) | 6,729 (6.6) | 20,972 (8.4) |  |
| **Maternal smoking at registration, n (%)** | | | | | | | | | | | | | | | | | |
| Non-smoking | 51,596 (85.8) | 106,549 (86.1) | 6,992 (88.9) | 16,032 (90.0) | 8,759 (88.0) | 44,743 (89.5) | 67,347 (86.4) | 167,324 (87.3) | 65,815 (84.4) | 140,228 (84.6) | 6,314 (86.8) | 12,577 (87.8) | 15,045 (87.2) | 61,988 (88.2) | 87,174 (85.1) | 214,793 (85.8) |  |
| 1–9 cig/day | 4,963 (8.3) | 9,482 (7.7) | 527  (6.7) | 1,030  (5.8) | 651  (6.5) | 2,782  (5.6) | 6,141  (7.9) | 13,294  (6.9) | 5,541  (7.1) | 11,168  (6.7) | 459  (6.3) | 792  (5.5) | 1,034  (6.0) | 3,461  (4.9) | 7,034 (6.9) | 15,421 (6.2) |  |
| 10+ cig/day | 1,991 (3.3) | 4,030 (3.3) | 156  (2.0) | 323  (1.8) | 198  (2.0) | 802  (1.6) | 2,345  (3.0) | 5,155  (2.7) | 1,909  (2.4) | 4,064  (2.5) | 101  (1.4) | 173  (1.2) | 320  (1.9) | 935  (1.3) | 2,330 (2.3) | 5,172 (2.1) |  |
| Missing | 1,591 (2.6) | 3,733 (3.0) | 194  (2.5) | 426  (2.4) | 344  (3.5) | 1,664  (3.3) | 2,129  (2.7) | 5,823  (3.0) | 4,701  (6.0) | 10,223  (6.2) | 398  (5.5) | 782  (5.5) | 853  (4.9) | 3,936  (5.6) | 5,952 (5.8) | 14,941 (6.0) |  |
| **Maternal smoking at gestation weeks 30-32, n (%)** | | | | | | | | | | | | | | | | | |
| Non-smoking | 49,093 (81.6) | 97,051 (78.4) | 7,034 (89.4) | 16,114 (90.5) | 8,789 (88.3) | 44,918 (89.9) | 64,916 (83.3) | 158,083 (82.5) | 64,657 (82.9) | 131,842 (79.6) | 6,406 (88.1) | 12,737 (88.9) | 15,340 (88.9) | 62,979 (89.6) | 86,403 (84.3) | 207,558 (82.9) |  |
| 1–9 cig/day | 3,584 (6.0) | 6,325 (5.1) | 380  (4.8) | 745  (4.2) | 459  (4.6) | 2,007  (4.0) | 4,423  (5.7) | 9,077  (4.7) | 3,887  (5.0) | 7,450  (4.5) | 365  (5.0) | 623  (4.3) | 789  (4.6) | 2,669  (3.8) | 5,041 (4.9) | 10,742 (4.3) |  |
| 10+ cig/day | 1,285 (2.1) | 2,358 (1.9) | 99  (1.3) | 210  (1.2) | 129  (1.3) | 544  (1.1) | 1,513  (1.9) | 3,112  (1.6) | 1,295  (1.7) | 2,495  (1.5) | 81  (1.1) | 138  (1.0) | 216  (1.3) | 645  (0.9) | 1,592 (1.6) | 3,278 (1.3) |  |
| Missing | 6,179 (10.3) | 18,060 (14.6) | 356  (4.5) | 742  (4.2) | 575  (5.8) | 2,522  (5.0) | 7,110  (9.1) | 21,324  (11.1) | 8,127  (10.4) | 23,896  (14.4) | 420  (5.8) | 826  (5.8) | 907  (5.3) | 4,027  (5.7) | 9,454 (9.2) | 28,749 (11.5) |  |

PCV = pneumococcal conjugate vaccine, PCV7/PCV13 = 7-valent/13-valent pneumococcal conjugate vaccine, PHiD-CV = pneumococcal non-typeable *Haemophilus influenzae* protein D conjugate vaccine, N = number of children in the specified cohort, n (%) = number (percentage) of children, SD = standard deviation, cig = cigarette, OM = children with one or more otitis media/acute otitis media diagnosis within the study period, All = full population regardless of otitis media/acute otitis media diagnosis, VGR = Västra Götalandsregionen

Table S4 Frequency of comorbidities in children

| **Skåne** | | | | | | | | | | | | | | | | | | | **VGR** | | | | | | | | | | | | | | | |
| --- | --- | --- | --- | --- | --- | --- | --- | --- | --- | --- | --- | --- | --- | --- | --- | --- | --- | --- | --- | --- | --- | --- | --- | --- | --- | --- | --- | --- | --- | --- | --- | --- | --- | --- |
| **pre-PCV n (%)** | | | **PCV7 n (%)** | | | | | **PHiD-CV n (%)** | | | | | | **Overall n (%)** | | | | | **pre-PCV n (%)** | | | | **PCV7 n (%)** | | | | **PCV13 n (%)** | | | | **Overall n (%)** | | | |
| **OM** | **All** | | **OM** | | **All** | | | **OM** | | | **All** | | | **OM** | | | **All** | | **OM** | | **All** | | **OM** | | **All** | | **OM** | | **All** | | **OM** | | **All** | |
| **Congenital/acquired immunodeficiency*** | | | | | | | | | | | | | | | | | | | | | | | | | | | | | | | | | | |
| 363  (0.6) | 562  (0.5) | | 33  (0.4) | | 51  (0.3) | | | 31  (0.3) | | | 57  (0.1) | | | 427  (0.5) | | | 670  (0.3) | | 103  (0.1) | | 179  (0.1) | | 10  (0.1) | | 13  (0.1) | | 11  (0.1) | | 28  (0.0) | | 124  (0.1) | | 220  (0.1) | |
| **Human immunodeficiency virus infection*** | | | | | | | | | | | | | | | | | | | | | | | | | | | | | | | | | | |
| 12  (0.0) | 16  (0.0) | | 1  (0.0) | | | 1  (0.0) | | | 1  (0.0) | | | 2  (0.0) | | | 14  (0.0) | | | 19  (0.0) | | 3  (0.0) | | 7  (0.0) | | 2  (0.0) | | 3  (0.0) | | 1  (0.0) | | 4  (0.0) | | 6  (0.0) | | 14  (0.0) |
| **Chronic renal failure*** | | | | | | | | | | | | | | | | | | | | | | | | | | | | | | | | | | |
| 17  (0.0) | 32  (0.0) | | 3  (0.0) | | | 4  (0.0) | | | 3  (0.0) | | | 9  (0.0) | | | 23  (0.0) | | | 45  (0.0) | | 26  (0.0) | | 49  (0.0) | | 1  (0.0) | | 3  (0.0) | | 1  (0.0) | | 7  (0.0) | | 28  (0.0) | | 59  (0.0) |
| **Nephrotic syndrome*** | | | | | | | | | | | | | | | | | | | | | | | | | | | | | | | | | | |
| 29  (0.0) | 49  (0.0) | | 1  (0.0) | | | 3  (0.0) | | | 1  (0.0) | | | 2  (0.0) | | | 31  (0.0) | | | 54  (0.0) | | 38  (0.0) | | 69  (0.0) | | 2  (0.0) | | 4  (0.0) | | 4  (0.0) | | 5  (0.0) | | 44  (0.0) | | 78  (0.0) |
| **Generalised malignancy*, Leukemia*, Lymphoma*, Hodgkin disease*, Multiple myeloma*** | | | | | | | | | | | | | | | | | | | | | | | | | | | | | | | | | | |
| 129  (0.2) | 238  (0.2) | | 6  (0.1) | | | 15  (0.1) | | | 10  (0.1) | | | 29  (0.1) | | | 145  (0.2) | | | 282  (0.1) | | 176  (0.2) | | 340  (0.2) | | 15  (0.2) | | 20  (0.1) | | 14  (0.1) | | 46  (0.1) | | 205  (0.2) | | 406  (0.2) |
| **Iatrogenic immunosuppression*** | | | | | | | | | | | | | | | | | | | | | | | | | | | | | | | | | | |
| 92  (0.2) | 128  (0.1) | | 5  (0.1) | | | 13  (0.1) | | | 8  (0.1) | | | 16  (0.0) | | | 105  (0.1) | | | 157  (0.1) | | 145  (0.2) | | 228  (0.1) | | 14  (0.2) | | 20  (0.1) | | 13  (0.1) | | 26  (0.0) | | 172  (0.2) | | 274  (0.1) |
| **Solid organ transplant*** | | | | | | | | | | | | | | | | | | | | | | | | | | | | | | | | | | |
| 14  (0.0) | 21  (0.0) | | 1  (0.0) | | | 2  (0.0) | | | 3  (0.0) | | | 7  (0.0) | | | 18  (0.0) | | | 30  (0.0) | | 18  (0.0) | | 2  (0.0) | | 2  (0.0) | | 2  (0.0) | | 1  (0.0) | | 2  (0.0) | | 21  (0.0) | | 31  (0.0) |
| **Functional or anatomic asplenia** | | | | | | | | | | | | | | | | | | | | | | | | | | | | | | | | | | |
| 15  (0.0) | 22  (0.0) | | 2  (0.0) | | | 3  (0.0) | | | 1  (0.0) | | | 6  (0.0) | | | 18  (0.0) | | | 31  (0.0) | | 14  (0.0) | | 29  (0.0) | | 1  (0.0) | | 1  (0.0) | | 2  (0.0) | | 10  (0.0) | | 17  (0.0) | | 40  (0.0) |
| **Cerebrospinal fluid leak** | | | | | | | | | | | | | | | | | | | | | | | | | | | | | | | | | | |
| 3  (0.0) | 4  (0.0) | 0  (0.0) | | 0  (0.0) | | | 1  (0.0) | | | 2  (0.0) | | | 4  (0.0) | | | 6  (0.0) | | | 2  (0.0) | | 5  (0.0) | | 0  (0.0) | | 0  (0.0) | | 0  (0.0) | | 1  (0.0) | | 2  (0.0) | | 6  (0.0) | |
| **Cochlear implant** | | | | | | | | | | | | | | | | | | | | | | | | | | | | | | | | | | |
| 31  (0.1) | 42  (0.0) | 6  (0.1) | | 7  (0.0) | | | 3  (0.0) | | | 9  (0.0) | | | 40  (0.1) | | | 58  (0.0) | | | 23  (0.0) | | 36  (0.0) | | 5  (0.1) | | 5  (0.0) | | 7  (0.0) | | 12  (0.0) | | 35  (0.0) | | 53  (0.0) | |
| **Heart disease** | | | | | | | | | | | | | | | | | | | | | | | | | | | | | | | | | | |
| 1,283  (2.1) | 2,198  (1.8) | 220  (2.8) | | 417  (2.3) | | | 273  (2.7) | | | 997  (2.0) | | | 1,776  (2.3) | | | 3,612  (1.9) | | | 1,683  (2.2) | | 3,029  (1.8) | | 157  (2.2) | | 293  (2.0) | | 412  (2.4) | | 1,502  (2.1) | | 2,252  (2.2) | | 4,824 (1.9) | |
| **Liver disease (chronic)** | | | | | | | | | | | | | | | | | | | | | | | | | | | | | | | | | | |
| 56  (0.1) | 85  (0.1) | 7  (0.1) | | 9  (0.1) | | | 3  (0.0) | | | 12  (0.0) | | | 66  (0.1) | | | 106  (0.1) | | | 55  (0.1) | | 107  (0.1) | | 5  (0.1) | | 6  (0.0) | | 10  (0.1) | | 32  (0.0) | | 70  (0.1) | | 145  (0.1) | |
| **Lung disease (chronic)** | | | | | | | | | | | | | | | | | | | | | | | | | | | | | | | | | | |
| 9,588 (15.9) | 15,275 (12.3) | 1,332 (16.9) | | 2,134 (12.0) | | | 1,326 (13.3) | | | 2,680 (5.4) | | | 12,246 (15.7) | | | 20,089 (10.5) | | | 9,807 (12.6) | | 15,787 (9.5) | | 934 (12.8) | | 1,438 (10.0) | | 1,864 (10.8) | | 3,554 (5.1) | | 12,605 (12.3) | | 20,779 (8.3) | |
| **Diabetes** | | | | | | | | | | | | | | | | | | | | | | | | | | | | | | | | | | |
| 263  (0.4) | 505  (0.4) | 8  (0.1) | | 19  (0.1) | | | 2  (0.0) | | | 16  (0.0) | | | 273  (0.4) | | | 540  (0.3) | | | 314  (0.4) | | 741  (0.4) | | 6  (0.1) | | 22  (0.2) | | 17  (0.1) | | 38  (0.1) | | 337  (0.3) | | 801  (0.3) | |

PCV = pneumococcal conjugate vaccine, PCV7/PCV13 = 7-valent/13-valent pneumococcal conjugate vaccine, PHiD-CV = pneumococcal non-typeable *Haemophilus influenzae* protein D conjugate vaccine, n (%) = number (percentage) of children, OM = children with one or more otitis media/acute otitis media diagnosis within the study period, All = full population regardless of otitis media/acute otitis media diagnosis, VGR = Västra Götalandsregionen.

*defined as immunocompromised

Table S5 Overall incidence per 100,000 person-years of severe otitis media/acute otitis media in the pre-PCV, PCV7, and PHiD-CV/PCV13 cohorts

| **Cohort** | **≤2 years** | **3–5 years** | **≤5 years** |
| --- | --- | --- | --- |
| **Skåne Incidence rate (95% CI)** | | | |
| pre-PCV | 5,601 (5,502; 5,702) | 1,957 (1,908; 2,006) | 3,446 (3,396; 3,496) |
| PCV7 | 4,276 (4,104; 4,455) | 1,900 (1,722; 2,097) | 3,609 (3,475; 3,749) |
| PHiD-CV | 2,791 (2,679; 2,907) | 1,691 (1,001; 2,855) | 2,780 (2,669; 2,896) |
| **VGR Incidence rate (95% CI)** | | | |
| pre-PCV | 3,620 (3,551; 3,691) | 1,231 (1,198; 1,265) | 2,201 (2,167; 2,236) |
| PCV7 | 3,424 (3,254; 3,604) | 802 (686; 936) | 2,595 (2,472; 2,724) |
| PCV13 | 2,465 (2,380; 2,552) | 1,154 (905; 1,472) | 2,409 (2,327; 2,494) |

CI = confidence interval, PCV = pneumococcal conjugate vaccine, PCV7/PCV13 = 7-valent/13-valent pneumococcal conjugate vaccine, PHiD-CV = pneumococcal non-typeable *Haemophilus influenzae* protein D conjugate vaccine, VGR = Västra Götalandsregionen

Table S6 Interrupted time-series analysis results for incidence of otitis media/acute otitis media

|  | Estimates (95% CI) | | | | | | | |
| --- | --- | --- | --- | --- | --- | --- | --- | --- |
|  | Comparison | <1 year | 1 year | 2 years | 3 years | 4 years | 5 years | ≤5 years |
| Skåne |  | | | | | | | |
| Change in slope | Pre-PCV - PCV7* | -492 (-1,108; 125) | -174 (-1,439; 1,092) | 3 (-781; 787) | -161 (-653; 330) | 74 (-395; 542) | -130 (-541; 282) | -102 (-665; 460) |
|  | Pre-PCV - PHiD-CV | **-365 (-692; -38)** | **-820 (-1,403; -237)** | **-552 (-904; -200)** | -197 (-404; 9) | -196 (-407; 14) | -125 (-329; 79) | **-382 (-641; -123)** |
|  | PCV7 - PHiD-CV* | 127 (-501; 754) | -646 (-1,931; 639) | -555 (-1,349; 240) | -36 (-537; 465) | -270 (-745; 205) | 4 (-414; 423) | -280 (-849; 290) |
| Change in level | Pre-PCV - PCV7* | -3,878 (-9,694; 1,937) | **-13,149 (-24,888; -1,409)** | **-8,935 (-15,905; -1,964)** | -1,986 (-6,271; 2,299) | -3,921 (-8,045; 203) | -4,273 (-8,084; -463) | **-6,143 (-11,140; -1,146)** |
|  | Pre-PCV - PHiD-CV | -6,073 (-15,547; 3,400) | **-19,056 (-36,298; -1,815)** | -8,424 (-18,773; 1,925) | -2,493 (-8,410; 3,424) | -1,594 (-7,722; 4,533) | -3,117 (-9,019; 2,786) | -6,539 (-14,032; 955) |
|  | PCV7 - PHiD-CV* | 6,164 (-743; 13,071) | -2,955 (-17,487; 11,577) | 463 (-8,279; 9,205) | 2,236 (-3,276; 7,748) | 1,075 (-4,122; 6,271) | 3,353 (-1,279; 7,985) | 1,346 (-4,910; 7,603) |
| VGR | | | | | | | | |
| Change in slope | Pre-PCV - PCV7* | -668 (-1,430; 95) | **-1,845 (-3,640; -49)** | -1,334 (-2,754; 86) | **-1,026 (-2,039; -12)** | **-922 (-1,787; -57)** | -222 (-941; 497) | **-1,019 (-1,938; -101)** |
|  | Pre-PCV - PCV13 | **-185 (-318; -51)** | **-825 (-1,246; -404)** | **-664 (-988; -341)** | **-425 (-657; -193)** | **-289 (-487; -91)** | **-197 (-363; -31)** | **-436 (-651; -221)** |
|  | PCV7 - PCV13* | 483 (-277; 1,244) | 1,020 (-774; 2,813) | 669 (-750; 2,089) | 601 (-413; 1,614) | 633 (-232; 1,498) | 24 (-695; 743) | 583 (-335; 1,501) |
| Change in level | Pre-PCV - PCV7* | -543 (-4,844; 3,757) | 603 (-10,004; 11,209) | -1,802 (-10,001; 6,398) | -1,044 (-6,883; 4,795) | -67 (-5,045; 4,910) | -2,613 (-6,772; 1,547) | -730 (-6,041; 4,582) |
|  | Pre-PCV - PCV13 | 1,134 (-2,743; 5,010) | 2,149 (-9,340; 13,638) | 3,878 (-4,952; 12,709) | 1,719 (-4,560; 7,998) | 545 (-4,810; 5,901) | 1,269 (-3,233; 5,771) | 1,951 (-3,801; 7,702) |
|  | PCV7 - PCV13* | **9,691 (2,774; 16,607)** | **23,684 (7,412; 39,957)** | **21,685 (8,966; 34,404)** | **15,074 (6,004; 24,144)** | **11,677 (3,936; 19,418)** | **6,537 (94; 12,980)** | **14,910 (6,682; 23,138)** |

CI = confidence interval, PCV = pneumococcal conjugate vaccine, PCV7/PCV13 = 7-valent/13-valent pneumococcal conjugate vaccine, PHiD-CV = pneumococcal non-typeable *Haemophilus influenzae* protein D conjugate vaccine, VGR = Västra Götalandsregionen. Bold indicates statistically significant changes.

*the PCV7 era was short in VGR and Skåne and any change detected vs the PCV7 period in the incidence of OM in the time-series analyses should be considered with caution

Table S 7 Sensitivity analysis: Interrupted time-series results for incidence of otitis media/acute otitis media using an extended transition period which spanned the introduction of both PCV13 and PHiD-CV

|  | Estimates (95% CI) | | | | | | | |
| --- | --- | --- | --- | --- | --- | --- | --- | --- |
|  | Comparison | <1 year | 1 year | 2 years | 3 years | 4 years | 5 years | ≤5 years |
| Skåne |  | | | | | | | |
| Change in slope | Pre-PCV - PCV7* | **-1,666 (-2,548; -784)** | **-2,911 (-5,222; -599)** | **-1,665 (-3,034; -295)** | **-1,254 (-2,251; -257)** | -486 (-1,460; 488) | -319 (-1,099; 461) | **-1,466 (-2,555; -378)** |
|  | Pre-PCV - PHiD-CV | **-282 (-481; -82)** | **-717 (-1,205; -229)** | **-500 (-801; -198)** | -168 (-342; 5) | -188 (-404; 28) | -126 (-330; 77) | **-344 (-574; -115)** |
|  | PCV7 - PHiD-CV* | **1,384 (479; 2,289)** | 2,193 (-183; 4,570) | 1,165 (-239; 2,569) | **1,086 (69; 2,104)** | 298 (-700; 1,296) | 193 (-613; 998) | **1,122 (3; 2,241)** |
| Change in level | Pre-PCV - PCV7* | 1,463 (-3,480; 6,407) | -1,339 (-14,222; 11,545) | -2,324 (-9,940; 5,291) | 2,384 (-2,733; 7,501) | -1,743 (-7,175; 3,688) | -3,425 (-8,048; 1,197) | -596 (-6,582; 5,391) |
|  | Pre-PCV - PHiD-CV | -5,073 (-10,796; 650) | **-18,713 (-32,935; -4,490)** | -8,051 (-16,761; 659) | -2,307 (-7,297; 2,683) | -1,694 (-7,934; 4,547) | -3,395 (-9,169; 2,379) | **-6,714 (-13,251; -176)** |
|  | PCV7 - PHiD-CV* | **21,776 (10,378; 33,174)** | **32,105 (2,150; 62,059)** | **22,570 (4,889; 40,251)** | **16,634 (3,580; 29,687)** | 8,307 (-4,278; 20,892) | 5,451 (-4,641; 15,544) | **18,811 (4,729; 32,894)** |
| VGR | | | | | | | | |
| Change in slope | Pre-PCV - PCV7* | **-1,130 (-2,022; -238)** | **-2,351 (-4,318; -384)** | **-1,709 (-3,194; -223)** | -994 (-2,080; 92) | **-980 (-1,915; -45)** | -172 (-938; 593) | **-1,185 (-2,163; -206)** |
|  | Pre-PCV - PCV13 | **-154 (-287; -21)** | **-778 (-1,214; -342)** | **-622 (-952; -291)** | **-436 (-677; -195)** | **-286 (-495; -77)** | **-208 (-380; -36)** | **-416 (-638; -194)** |
|  | PCV7 - PCV13* | **976 (65; 1,888)** | 1,573 (-450; 3,597) | 1,087 (-436; 2,610) | 558 (-556; 1,671) | 694 (-265; 1,653) | -37 (-822; 748) | 769 (-235; 1,772) |
| Change in level | Pre-PCV - PCV7* | 1,191 (-3,344; 5,726) | 2,672 (-8,422; 13,766) | -150 (-8,484; 8,184) | -1,195 (-7,269; 4,879) | 198 (-5,038; 5,435) | -2,831 (-7,130; 1,469) | 1 (-5,489; 5,491) |
|  | Pre-PCV - PCV13 | -346 (-4,392; 3,700) | -3,205 (-15,763; 9,352) | -574 (-10,179; 9,030) | -69 (-7,052; 6,914) | -1,029 (-7,064; 5,006) | 623 (-4,358; 5,603) | -765 (-7,126; 5,596) |
|  | PCV7 - PCV13* | **17,678 (5,888; 29,467)** | **34,091 (8,569; 59,614)** | **28,620 (9,427; 47,813)** | **18,027 (3,998; 32,056)** | **15,435 (3,364; 27,507)** | 6,372 (-3,526; 16,271) | **19,374 (6,740; 32,008)** |

CI = confidence interval, PCV = pneumococcal conjugate vaccine, PCV7/PCV13 = 7-valent/13-valent pneumococcal conjugate vaccine, PHiD-CV = pneumococcal non-typeable *Haemophilus influenzae* protein D conjugate vaccine, VGR = Västra Götalandsregionen. Bold indicates statistically significant changes.

*the PCV7 era was short in VGR and Skåne and any change detected vs the PCV7 period in the incidence of OM in the time-series analyses should be considered with caution

Table S 8 Sensitivity analysis: Estimating underlying otitis media/acute otitis media incidence allowing for vaccine coverage

| Region | Incidence | 2009 | 2010 | 2011 | 2012 | 2013 |
| --- | --- | --- | --- | --- | --- | --- |
| Skåne | Actual | 30,745 | 29,319 | 24,835 | 23,918 | 20,206 |
|  | Pre-PCV | 30,888 | 30,525 | 27,808 | 29,733 | 27,072 |
|  | Estimated | 30,742 | 29,292 | 24,772 | 23,781 | 20,020 |
| VGR | Actual | 26,453 | 31,172 | 25,670 | 24,227 | 21,411 |
|  | Pre-PCV | 26,579 | 32,685 | 30,196 | 32,623 | 31,389 |
|  | Estimated | 26,450 | 31,137 | 25,569 | 24,026 | 21,133 |

VGR = Västra Götalandsregionen.

‘Actual’ incidences are derived using officially reported annual coverage rates. There was no difference between the estimated and actual incidence rates for the years 2005–2008 (data not shown)

Table S9 Association between otitis media/acute otitis media (OM) diagnoses and covariates according to a Poisson model within each vaccine cohort (≤5 years of age)

|  | Skåne | | VGR | | Skåne | | VGR | | Skåne | | VGR | |
| --- | --- | --- | --- | --- | --- | --- | --- | --- | --- | --- | --- | --- |
|  | **Pre-PCV** | | **Pre-PCV** | | **PCV7** | | **PCV7** | | **PHiD-CV** | | **PCV13** | |
|  | **IRR**  **(95% CI)** | **p-value** | **IRR**  **(95% CI)** | **p-value** | **IRR**  **(95% CI)** | **p-value** | **IRR**  **(95% CI)** | **p-value** | **IRR**  **(95% CI)** | **p-value** | **IRR**  **(95% CI)** | **p-value** |
| Age (ref: <1 years) | | <0.001 |  | <0.001 |  | <0.001 |  | <0.001 |  | <0.001 |  | <0.001 |
| 1 year | 1.154  (1.111; 1.200) | <0.001 | 1.35  (1.292; 1.411) | <0.001 | 0.924  (0.853; 1.000) | 0.050 | 1.305  (1.157; 1.473) | <0.001 | 1.142  (1.065; 1.225) | <0.001 | 1.200  (1.126; 1.279) | <0.001 |
| 2 years | 0.564  (0.539; 0.590) | <0.001 | 0.779  (0.741; 0.820) | <0.001 | 0.599  (0.532; 0.675) | <0.001 | 0.809  (0.660; 0.992) | 0.042 | 0.563  (0.507; 0.625) | <0.001 | 0.598  (0.547; 0.653) | <0.001 |
| 3 years | 0.361  (0.343; 0.381) | <0.001 | 0.506  (0.478; 0.536) | <0.001 | 0.484  (0.410; 0.572) | <0.001 | 0.642  (0.477; 0.864) | 0.004 | 0.376  (0.270; 0.524) | <0.001 | 0.383  (0.322; 0.456) | <0.001 |
| 4 years | 0.299  (0.282; 0.317) | <0.001 | 0.384  (0.361; 0.410) | <0.001 | 0.422  (0.336; 0.531) | <0.001 | 0.572  (0.389; 0.840) | 0.005 | - | - | - | - |
| 5 years | 0.215  (0.200; 0.230) | <0.001 | 0.3  (0.279; 0.322) | <0.001 | - | - | - | - | - | - | - | - |
| Sex (ref: Male) | |  |  |  |  |  |  |  |  |  |  |  |
| Female | 0.912  (0.892; 0.934) | <0.001 | 0.92  (0.898; 0.943) | <0.001 | 0.895  (0.856; 0.937) | <0.001 | 0.879  (0.830; 0.932) | <0.001 | 0.829  (0.783; 0.877) | <0.001 | 0.867  (0.824; 0.912) | <0.001 |
| Past OM diagnoses (ref: none) | | <0.001 |  | <0.001 |  | <0.001 |  | <0.001 |  | <0.001 |  | <0.001 |
| One | 2.944  (2.845; 3.046) | <0.001 | 2.749  (2.654; 2.848) | <0.001 | 3.360  (3.156; 3.577) | <0.001 | 2.763  (2.553; 2.990) | <0.001 | 4.645  (4.306; 5.011) | <0.001 | 3.644  (3.405; 3.900) | <0.001 |
| Two or more | 6.73  (6.533; 6.934) | <0.001 | 5.632  (5.457; 5.812) | <0.001 | 7.539  (7.126; 7.976) | <0.001 | 5.545  (5.154; 5.965) | <0.001 | 9.965  (9.251; 10.734) | <0.001 | 7.462  (6.974; 7.984) | <0.001 |
| Month (ref: January) | | <0.001 |  | <0.001 |  | <0.001 |  | <0.001 |  | <0.001 |  | <0.001 |
| February | 1.438  (1.372; 1.507) | <0.001 | 1.365  (1.297; 1.437) | <0.001 | 1.496  (1.353; 1.654) | <0.001 | 1.510  (1.327; 1.717) | <0.001 | 1.209  (1.060; 1.378) | 0.005 | 1.197  (1.067; 1.343) | 0.002 |
| March | 1.335  (1.274; 1.398) | <0.001 | 1.398  (1.330; 1.470) | <0.001 | 1.408  (1.275; 1.554) | <0.001 | 1.519  (1.338; 1.724) | <0.001 | 1.018  (0.892; 1.161) | 0.796 | 1.082  (0.966; 1.212) | 0.174 |
| April | 1.003  (0.954; 1.055) | 0.898 | 1.061  (1.006; 1.120) | 0.031 | 1.038  (0.934; 1.153) | 0.492 | 1.204  (1.053; 1.377) | 0.007 | 0.977  (0.857; 1.115) | 0.734 | 0.953  (0.848; 1.071) | 0.417 |
| May | 0.871  (0.827; 0.917) | <0.001 | 0.897  (0.848; 0.949) | <0.001 | 0.945  (0.849; 1.051) | 0.296 | 1.033  (0.900; 1.186) | 0.642 | 0.811  (0.709; 0.929) | 0.003 | 0.779  (0.691; 0.878) | <0.001 |
| June | 0.706  (0.668; 0.747) | <0.001 | 0.703  (0.661; 0.747) | <0.001 | 0.678  (0.603; 0.761) | <0.001 | 0.813  (0.701; 0.943) | 0.007 | 0.607  (0.525; 0.702) | <0.001 | 0.609  (0.536; 0.692) | <0.001 |
| July | 0.353  (0.329; 0.379) | <0.001 | 0.331  (0.306; 0.358) | <0.001 | 0.370  (0.322; 0.425) | <0.001 | 0.402  (0.335; 0.484) | <0.001 | 0.360  (0.305; 0.426) | <0.001 | 0.332  (0.286; 0.387) | <0.001 |
| August | 0.363  (0.338; 0.390) | <0.001 | 0.336  (0.311; 0.364) | <0.001 | 0.425  (0.372; 0.487) | <0.001 | 0.440  (0.367; 0.527) | <0.001 | 0.327  (0.276; 0.388) | <0.001 | 0.300  (0.257; 0.351) | <0.001 |
| September | 0.704  (0.664; 0.745) | <0.001 | 0.706  (0.663; 0.751) | <0.001 | 0.744  (0.663; 0.835) | <0.001 | 0.881  (0.760; 1.021) | 0.093 | 0.567  (0.491; 0.655) | <0.001 | 0.552  (0.485; 0.628) | <0.001 |
| October | 0.789  (0.747; 0.834) | <0.001 | 0.831  (0.783; 0.882) | <0.001 | 0.885  (0.793; 0.987) | 0.028 | 0.982  (0.851; 1.133) | 0.801 | 0.705  (0.616; 0.807) | <0.001 | 0.683  (0.605; 0.770) | <0.001 |
| November | 0.858  (0.812; 0.907) | <0.001 | 0.939  (0.886; 0.995) | 0.034 | 0.980  (0.880; 1.091) | 0.709 | 1.119  (0.974; 1.286) | 0.112 | 0.802  (0.703; 0.915) | 0.001 | 0.765  (0.680; 0.860) | <0.001 |
| December | 0.985  (0.935; 1.038) | 0.574 | 1.077  (1.018; 1.138) | 0.009 | 1.059  (0.954; 1.175) | 0.285 | 1.112  (0.971; 1.275) | 0.125 | 0.916  (0.807; 1.040) | 0.176 | 0.885  (0.791; 0.990) | 0.033 |
| Time trend |  |  |  |  |  |  |  |  |  |  |  |  |
| Change per annum | 1.010  (0.995; 1.025) | 0.210 | 1.019  (1.001; 1.037) | 0.042 | 5.605  (4.380; 7.173) | <0.001 | 293.876  (104.497; 826.467) | <0.001 | 0.916  (0.876; 0.957) | <0.001 | 0.965  (0.932; 1.000) | 0.049 |
| Incremental change since PCV7 | 0.897  (0.863; 0.932) | <0.001 | 1.036  (0.983; 1.092) | 0.182 | - | - | - | - | - | - | - | - |
| Incremental change since PHiD-CV/PCV13 | 1.071  (1.025; 1.120) | 0.002 | 0.866  (0.821; 0.914) | <0.001 | 0.134  (0.103; 0.175) | <0.001 | 0.003  (0.001; 0.007) | <0.001 | - | - | - | - |

IRR = incidence rate ratio, CI = confidence interval, PCV7/PCV13 = 7-valent/13-valent pneumococcal conjugate vaccine, PHiD-CV = pneumococcal non-typeable *Haemophilus influenzae* protein D conjugate vaccine, VGR = Västra Götalandsregionen

Table S10 Adjusted* hazard ratios of time-to-first diagnosis of otitis media/acute otitis media in children ≤2 years of age

|  | Skåne | | | VGR | | |
| --- | --- | --- | --- | --- | --- | --- |
| PCV Cohorts | **HR** | **95% CI** | **p-value** | **HR** | **95% CI** | **p-value** |
| PHiD-CV/PCV13 relative to pre-PCV | 0.673 | 0.654; 0.692 | **<0.001** | 0.867 | 0.849; 0.886 | **<0.001** |
| PHiD-CV/PCV13 relative to PCV7 | 0.849 | 0.820; 0.879 | **<0.001** | 0.870 | 0.843; 0.898 | **<0.001** |
| PCV7 relative to pre-PCV | 0.792 | 0.771; 0.814 | **<0.001** | 0.997 | 0.969; 1.025 | 0.821 |

CI = confidence interval, HR = hazard ratio, PCV7/PCV13 = 7-valent/13-valent pneumococcal conjugate vaccine, PHiD-CV = pneumococcal non-typeable *Haemophilus influenzae* protein D conjugate vaccine, VGR = Västra Götalandsregionen.

*adjusted for sex, health condition at birth, maternal age, and maternal smoking at pre-conception and at 30 weeks

Figure S 1 Interrupted time-series fit of the monthly incidence of otitis media/acute otitis media (OM) in Skåne (A) and sensitivity analysis using an extended transition period which spanned the introduction of both high valency PCVs (B)


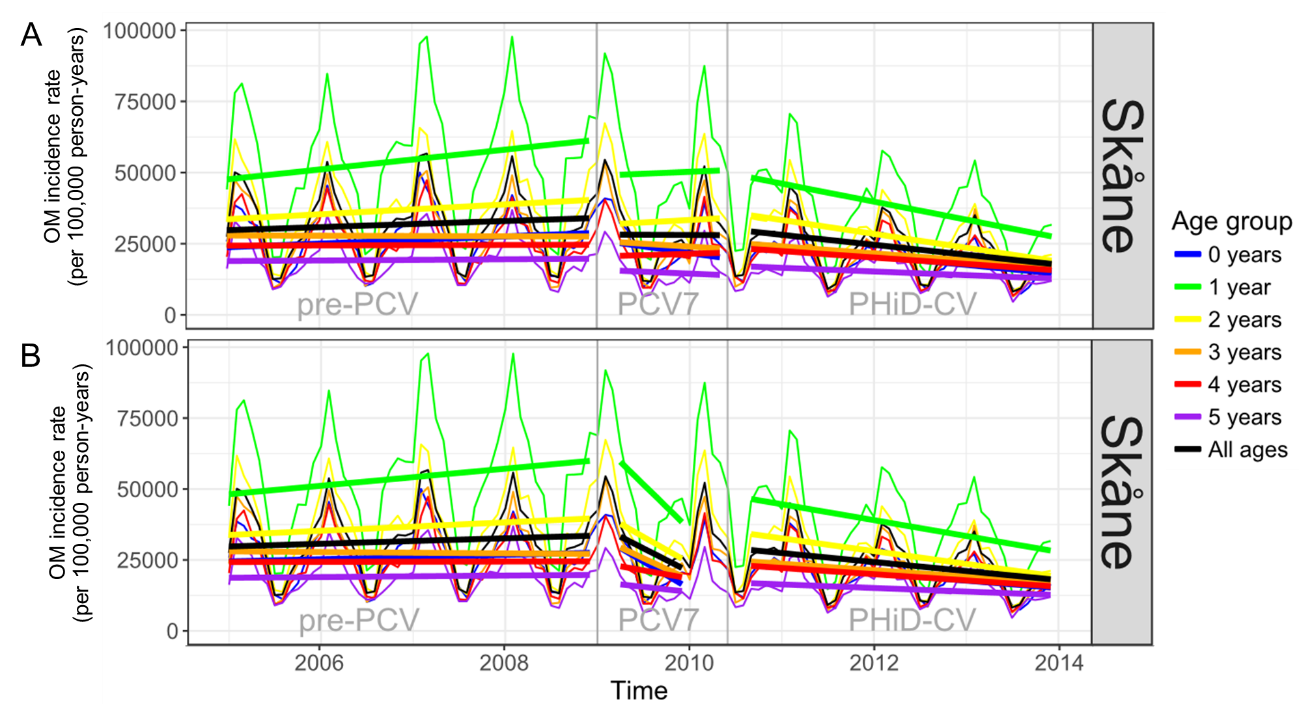


PCV = pneumococcal conjugate vaccine, PCV7 = 7-valent pneumococcal conjugate vaccine, PHiD-CV = pneumococcal non-typeable *Haemophilus influenzae* protein D conjugate vaccine

Figure S2 Interrupted time-series fit of the monthly incidence of otitis media/acute otitis media (OM) in VGR (A) and sensitivity analysis using an extended transition period which spanned the introduction of both high valency PCVs (B)


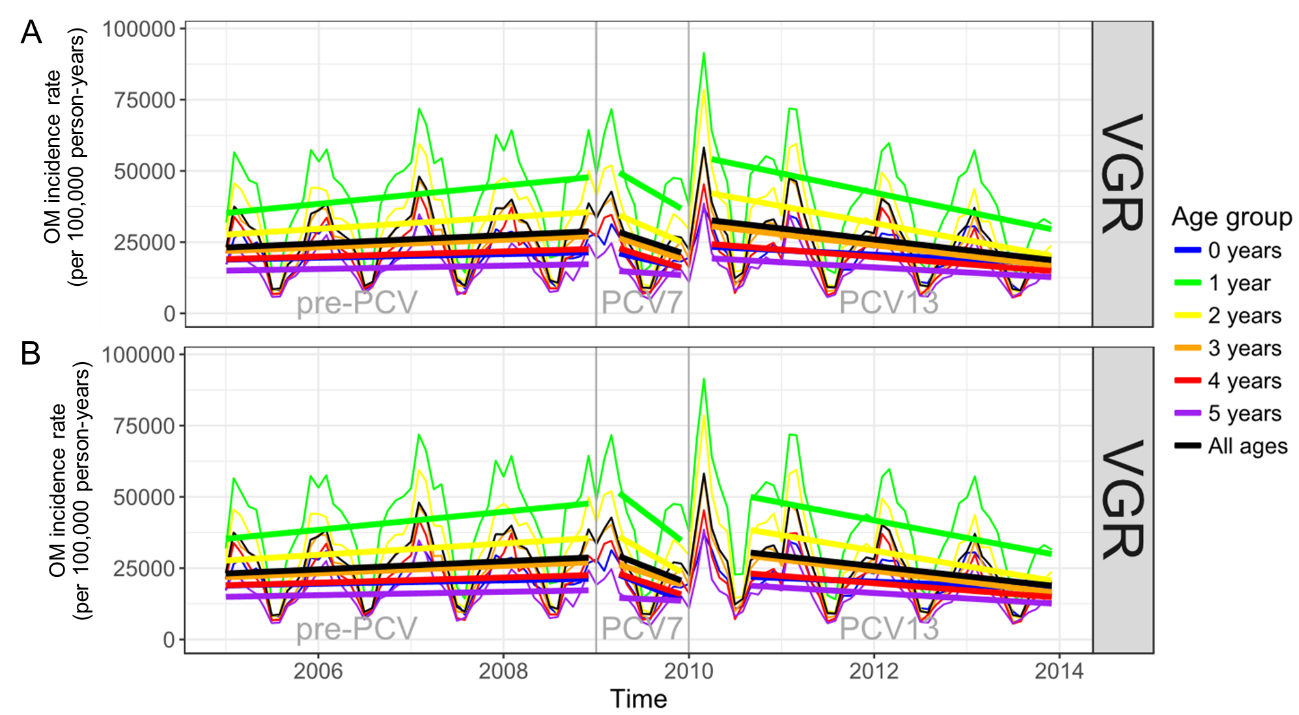


PCV = pneumococcal conjugate vaccine, PCV7/PCV13 = 7-valent/13-valent pneumococcal conjugate vaccine, VGR = Västra Götalandsregionen

Figure S3 Interrupted time-series fit of the monthly incidence of severe otitis media/acute otitis media (OM) in Skåne


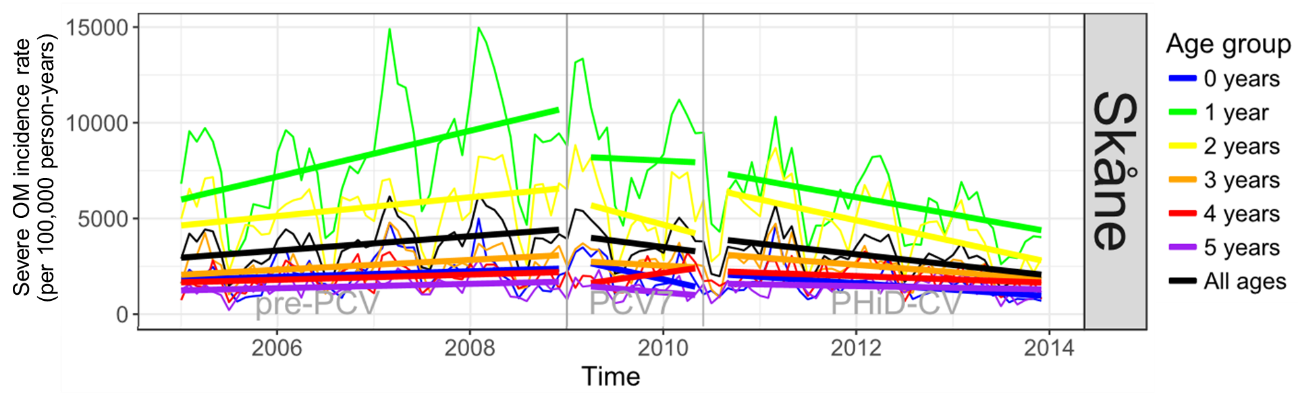


PCV = pneumococcal conjugate vaccine, PCV7 = 7-valent pneumococcal conjugate vaccine, PHiD-CV = pneumococcal non-typeable *Haemophilus influenzae* protein D conjugate vaccine

Figure S4 Interrupted time-series fit of the monthly incidence of severe otitis media/acute otitis media (OM) in VGR


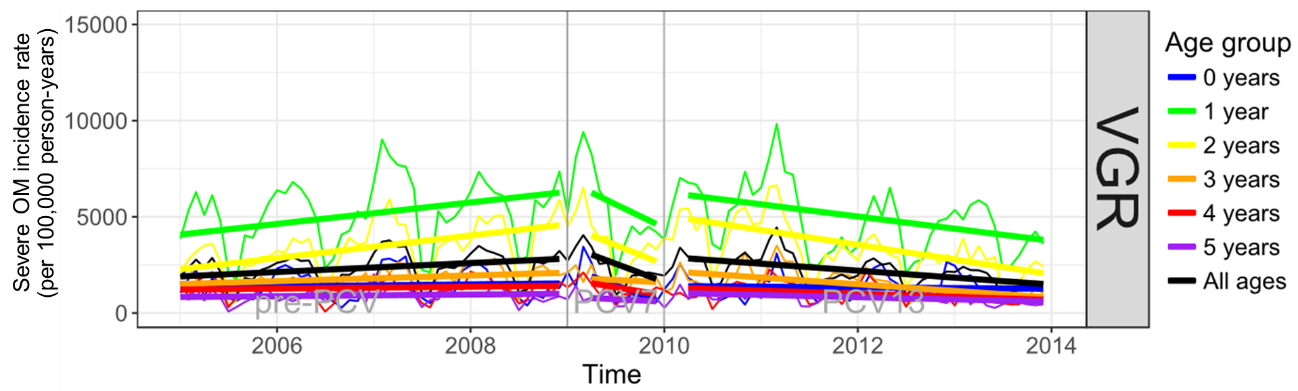


PCV = pneumococcal conjugate vaccine, PCV7/PCV13 = 7-valent/13-valent pneumococcal conjugate vaccine, VGR = Västra Götalandsregionen

Figure S5 Time-to-first diagnosis of otitis media/acute otitis media (OM) in children ≤2 years of age in Skåne


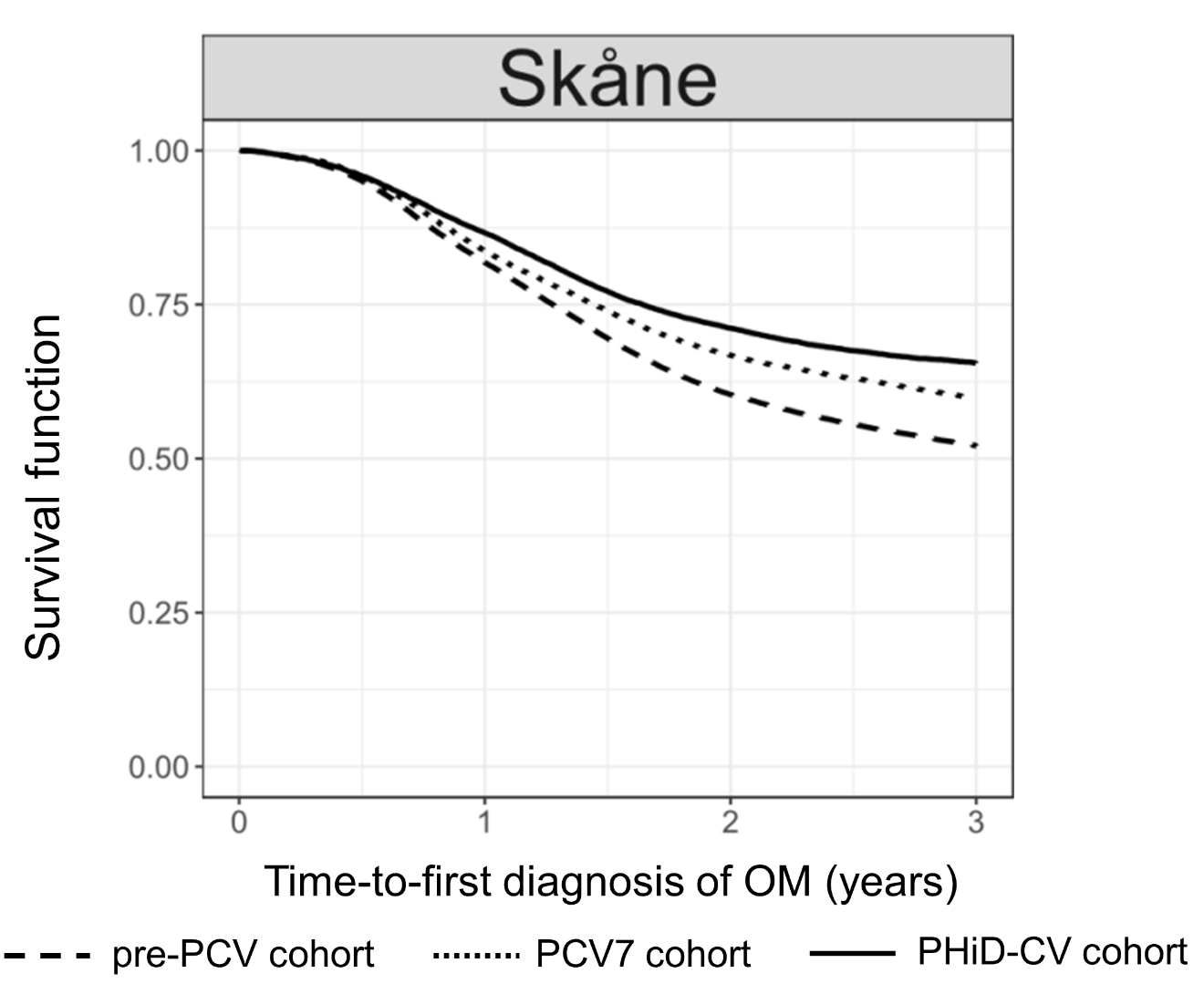


PCV = pneumococcal conjugate vaccine, PCV7 = 7-valent pneumococcal conjugate vaccine, PHiD-CV = pneumococcal non-typeable *Haemophilus influenzae* protein D conjugate vaccine

Figure S6 Time-to-first diagnosis of otitis media/acute otitis media (OM) in children ≤2 years of age in VGR


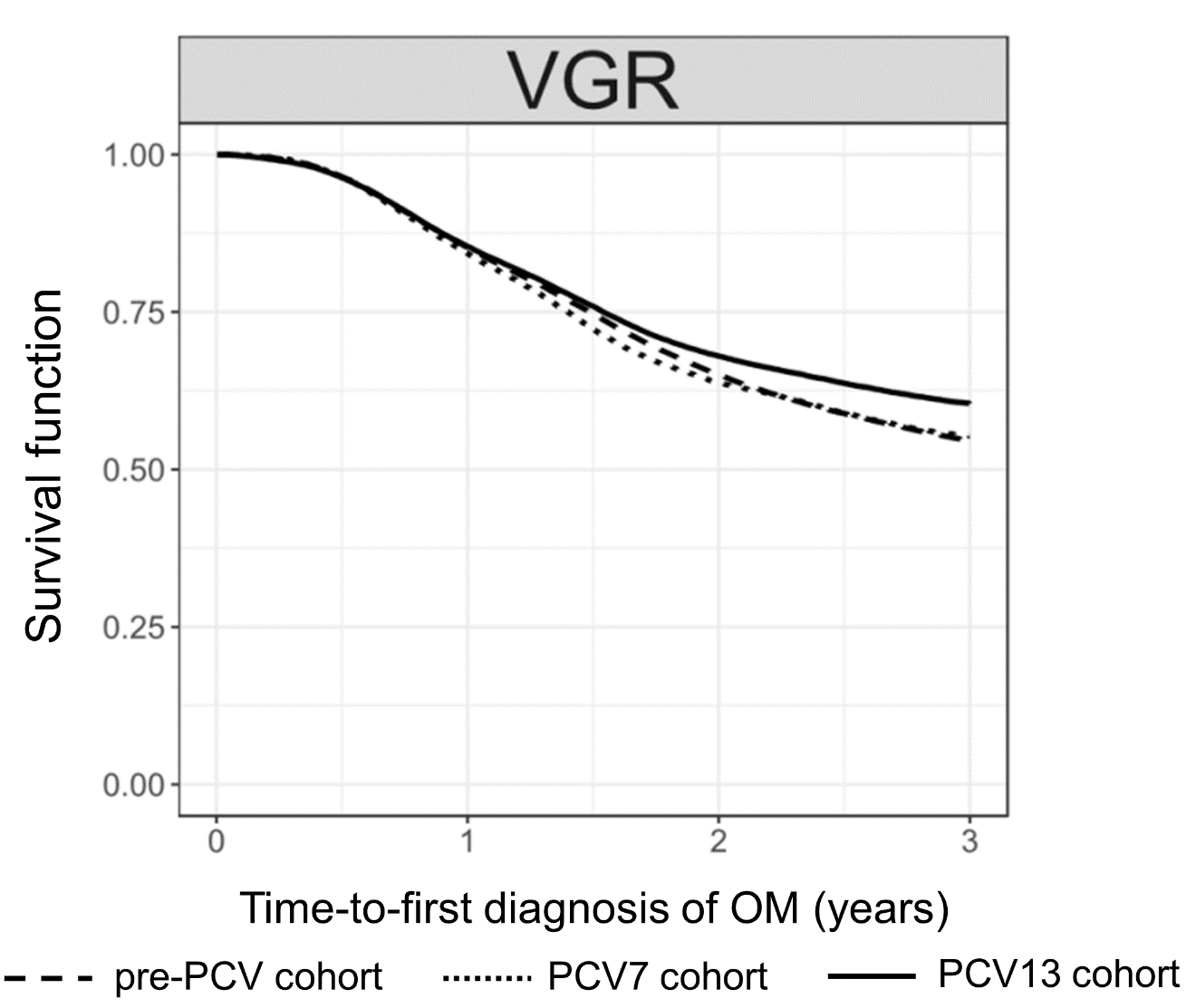


PCV = pneumococcal conjugate vaccine, PCV7/PCV13 = 7-valent/13-valent pneumococcal conjugate vaccine, VGR = Västra Götalandsregionen
